# Open-Celled Foams of Polyethersulfone/Poly(*N*-vinylpyrrolidone) Blends for Ultrafiltration Applications

**DOI:** 10.3390/polym14061177

**Published:** 2022-03-15

**Authors:** Aniket Raje, Kristian Buhr, Joachim Koll, Jelena Lillepärg, Volker Abetz, Ulrich A. Handge

**Affiliations:** 1 Institute of Membrane Research, Helmholtz-Zentrum Hereon, Max-Planck-Strasse 1, 21502 Geesthacht, Germany; aniket.raje@hereon.de (A.R.); kristian.buhr@hereon.de (K.B.); joachim.koll@hereon.de (J.K.); jelena.lillepaerg@hereon.de (J.L.); volker.abetz@hereon.de (V.A.); 2 Institute of Physical Chemistry, Universität Hamburg, Grindelallee 117, 20146 Hamburg, Germany; 3 Chair of Plastics Technology, Faculty of Mechanical Engineering, TU Dortmund University, Leonhard-Euler-Straße 5, 44227 Dortmund, Germany

**Keywords:** polymer membranes, open-celled foams, solvent-free membrane fabrication, polyethersulfone, ultrafiltration

## Abstract

Since membranes made of open porous polymer foams can eliminate the use of organic solvents during their manufacturing, a series of previous studies have explored the foaming process of various polymers including polyethersulfone (PESU) using physical blowing agents but failed to produce ultrafiltration membranes. In this study, blends containing different ratios of PESU and poly(*N*-vinylpyrrolidone) (PVP) were used for preparation of open-celled polymer foams. In batch foaming experiments involving a combination of supercritical CO_2_ and superheated water as blowing agents, blends with low concentration of PVP delivered uniform open-celled foams that consisted of cells with average cell size less than 20 µm and cell walls containing open pores with average pore size less than 100 nm. A novel sample preparation method was developed to eliminate the non-foamed skin layer and to achieve a high porosity. Flat sheet membranes with an average cell size of 50 nm in the selective layer and average internal pore size of 200 nm were manufactured by batch foaming a PESU blend with higher concentration of PVP and post-treatment with an aqueous solution of sodium hypochlorite. These foams are associated with a water-flux up to 45 L/(h m^2^ bar). Retention tests confirmed their applicability as ultrafiltration membranes.

## 1. Introduction

Ultrafiltration is an established membrane separation technique that is implemented to filter out nanoparticles by means of size exclusion or particle capture [1]. Ultrafiltration is used to purify liquids, e.g., water, whey, poly(vinyl alcohol) [2]. Porous polymeric membranes for ultrafiltration are typically manufactured using processes such as the non-solvent induced and thermally induced phase separation process (NIPS and TIPS, respectively) [3,4,5,6,7]. These processes involve organic solvents such as *N,N*-dimethylacetamide (DMAc), *N*-methyl-2-pyrrolidone (NMP), formic acid and *N,N*-dimethylformamide (DMF) and are classified as harmful chemicals [8,9,10,11]. Some of them are associated with liver disease in human beings [12]. The contamination of environmental water supply due to the disposal of organic solvents poses a serious risk. On-site incinerations are a common practice due to economic reasons [13]. To avoid wastage of organic solvents, in membrane industry it is a general practice to recirculate organic solvents using distillation, which consumes high amounts of energy leading to increased carbon emissions [14,15].

Over the past decades, polymer foaming is seen as a possible alternative to these organic solvent-based processes for fabrication of membranes. Various studies use the discontinuous process of batch foaming or solid state foaming [16]. Batch foaming involves a two-stage process where a physical gaseous blowing agent such as carbon dioxide (CO_2_) diffuses into a polymer at elevated temperature and pressure for a limited period. The temperature is generally below the glass transition temperature of the gas-loaded polymer causing it to remain in a so-called “solid state”. After completion of this stage, this polymer is exposed to a higher temperature for a short time, which causes nucleation and expansion of foam cells by taking advantage of the softened polymer. This creates a closed-celled or open-celled foam structure depending on the polymer properties and processing conditions. Open-celled polymer foams, when customized to desirable cell size, deliver a permeance that enables them to be implemented as separation membranes. As the aim of this work is to produce prototype ultrafiltration membranes without the use of organic solvents, we develop a manufacturing process using the batch foaming technique.

Krause et al. [17] produced closed-cellular polymer foams with an average pore diameter in the micrometer range (~100 µm) from PSU. After preparation of the said foam, they used the organic solvent tetrahydrofuran (THF) to form open pores in the nanometer range within the walls of the micro-sized cells. Krause et al. [18] used discontinuous solid state foaming of PSU/polyimide blends with CO_2_ as physical blowing agent to develop nanocellular foams. Microcellular open-cellular foams (diameter in the range of 1–10 µm) were achieved by the use of organic solvents. Nanocellular foams (range of pore diameter 2–50 nm) were achieved by increasing the CO_2_ saturation levels such that CO_2_ stays in a continuous phase, which leads to an open-celled structure. Although these foams find potential application as ultrafiltration membranes, their production involved the use of an organic solvent to achieve open pores smaller than 1 µm. Similarly, Gong et al. [19] produced porous cell walls in microcellular polycarbonate foams by using acetone with CO_2_ during foaming. The use of acetone induced crazing within the polymer thus resulting in a porous structure on the microcell walls. Without the use of organic solvents, Sorrentino et al. [20] and Guo et al. [21,22,23] developed foams with a similar structure. They investigated the foaming of high performance polymers including PESU and found that PESU exhibited nanocellular foams whereas the other investigated polymers exhibited pores with a diameter only in the micrometer range. They reported the formation of a nano-structure on the cell walls of microcellular foam of PESU and polyetherimide (PEI) without the use of organic solvents. This nano-structure appeared to have a tendency towards making the cell walls of the microcellular foams partially porous with pores less than 1 µm in diameter. Guo et al. [21,22] applied a solid state CO_2_ foaming process on PSU and PPSU where they used low temperatures between −10 °C to 60 °C for loading the samples with CO_2_ without using organic solvents. They reported a similar nanoscale structure on the cell walls of the closed microcellular foams. They describe this structure as a ‘network of stretched struts’ that are nanoscale fibers formed due to stress-induced nucleation or spinodal decomposition, i.e., a biaxial tensile deformation caused by the expansion of cells. Although this structure appears uniformly distributed on the cells, it does not appear to be open-celled foam. To obtain open pores within these foams, a higher porosity would be desirable such that pores would be created within such a nanoscale structure due to high degree of stretching.

Li et al. [24] used assisted mold foaming to manufacture polysulfone foams with high expansion ratios. They applied mechanical pressure on CO_2_-loaded polysulfone samples while subjecting them to foaming temperatures. By using this method, high expansion ratios were obtained in the resulting foams. However, the cell size was above 15 µm and only a closed-cellular structure was achieved. 

CO_2_ has been established as an ideal blowing agent for delivering high porosity polymer foams [16,25,26]. In addition, CO_2_ in supercritical state provides better foaming results than gaseous CO_2_ in subcriticial phase [16,27]. Hu et al. [28] studied the use of ethanol as a co-blowing agent during the foaming of polysulfone (PSU) and poly(phenylene sulfone) (PPSU). The expansion ratio and the foaming temperature window of the foams were significantly increased due to the use of co-blowing agent. Owusu-Nkwantabisah et al. [29] used a combination of supercritical CO_2_ and superheated H_2_O to produce PESU foams and found a significantly increased level of porosity and interconnectivity between pores as compared to a PESU foam that was foamed by using only supercritical CO_2_. Schulze et al. [30] also achieved open-celled foams using block copolymers and CO_2_ as foaming agent in the presence of water during the loading process. Water in superheated state has a significantly reduced polarity such that it shows solvent properties [31]. Therefore, the usage of superheated water with supercritical CO_2_ (shH_2_O + scCO_2_) for foaming emerges to be promising. 

Polyarylsulfones such as polyethersulfone (PESU), polysulfone (PSU) and polyphenylsulfone (PPSU) have been widely studied for obtaining permeable foams [17,18,20,21,22,29]. PESU is widely used for membranes as it provides high thermal stability due to its high glass transition temperature, good chemical stability due to the presence of sulfonyl groups and ether linkages, and favorable structural stability due to the presence of two aromatic groups in the repeating unit [32]. PESU membranes are also preferred for ultrafiltration applications due to their capability to deliver reliable retention values and high porosity [33]. Some researchers used poly(*N*-vinylpyrrolidone) (PVP) as a water-soluble ‘pore-opener’ along with poly(arylsulfones) to produce ultrafiltration membranes from solutions in organic solvent [33,34,35,36,37,38,39,40]. Therefore, due to the proven application of PESU and PVP in ultrafiltration membranes, we use blends of PESU and PVP in this work. Shi et al. [41] used PMMA/PVDF blends to achieve highly porous nanocellular foams. The use of PVDF to form a miscible blend with PMMA was determined to increase the porosity and decrease cell size in both macro and micro cells. Therefore, we plan studies to confirm the miscibility of PESU and PVP, and observe the effect of polymer content on various material characteristics and foaming results.

Ultrafiltration requires an average pore diameter between 10 nm and 100 nm in the selective layer. Thus, open-cellular foams with cell size of approximately 100 nm are targeted. As PVP is soluble in a variety of commonly available compounds such as water and aqueous solutions of sodium hypochlorite (NaOCl), a permeable selective layer could be created through the dissolution of PVP. NaOCl is selected due to its proven suitability for post-processing of PESU/PVP membranes [33,35,39]. The outer surface in batch foams commonly contains cracks that occur due to the expansion of the sample during foaming. If the cell size of the foam cells were in the range of micrometers, these cracks would lead to functional failure as ultrafiltration membrane and deem the selective layer useless. Therefore, the cell size is also desired to be similar to the cell size of the selective layer such that flaws occurring on the surface during foaming can be easily compensated by the internal structure. We focus on manufacturing completely open nanocellular foams, which the previous studies [17,18,19,20,21,22,23,24,25,26,27,28,29,30,41,42,43] did not fully realize. In recent years, the research on polymer foams has shifted towards composites involving graphene and other nanoparticles to deliver highly porous microcellular and nanocellular foams [44,45,46,47,48,49]. However, this is out of the scope of this work, as we focus on obtaining the said foam from the pristine polymers. 

Batch foaming often yields a non-foamed outer layer after foaming due to fast diffusion of the blowing agent [21]. In order to employ these foams as membranes, an intuitive method is developed to avoid this non-foamed layer and at the same time improve the foam quality. We aim to achieve a complete open-cellular foam structure with a nanometer cell size by combination of batch foaming and post-treatment in inorganic solvents. The membranes prepared using this organic solvent-free method would be permeable to water and have retention values that are near to those of ultrafiltration membranes based on methods using organic solvent in their manufacture.

## 2. Materials

In this study, commercial grades of polyethersulfone and poly(*N*-vinylpyrrolidone) BASF Luvitec^®^ K 30 (PVP K 30) were used for blend preparation. The blends were obtained from BASF SE (Ludwigshafen, Germany). Two variants of PESU that varied in molecular weight were used viz., BASF Ultrason^®^ E 2010 (PESU E 2010) and BASF Ultrason^®^ E 3020 P (PESU E 3020 P). Although high molecular weight PVP such as PVP K 90 is used in the fabrication of ultrafiltration membranes, PVP K 30 (BASF Luvitec^®^ K 30 molecular weight around 40,000 Da [50]) is used here because CO_2_ shows a higher miscibility with low molecular weight PVP than with high molecular weight PVP [51]. The blend formulations and nomenclature are given in Table 1.

BASF Luvitec^®^ K 90 (PVP K 90) and Kapton^®^ foil (Polyimide (PI) foil) was chosen for preparing sandwich-type samples. All materials were dried in a vacuum chamber at 130 °C for 24 h before further use. 

For post-treatment, sodium hypochlorite (Roth GmbH & Co. KG, Karlsruhe, Germany) and sodium bisulfate (Roth GmbH & Co. KG) were used.

## 3. Experimental and Methodology

### 3.1. Material Characterization

Differential scanning calorimetry (DSC) measurements were carried out using a calorimeter DSC 1 (Mettler Toledo, Gießen, Germany), and analyzed using STARe SW 16.20 software (Mettler Toledo). 40 μL aluminum pan with a mono-perforated lid was filled with approximately 10 mg of polymer. A heating rate of 10 K min^−1^ in the temperature interval from 25 °C to 260 °C in a nitrogen atmosphere was chosen. Heating-cooling-heating cycles were executed. Then the glass transition temperature was determined by analyzing the second heating interval. 

The glass transition temperatures and the heat capacities when changing from the glassy to the rubbery state of the homopolymers were used in the Equation (1) as derived by Couchman [52] and the glass transition temperatures were used in Equation (2) as derived by Fox [53] to find the expected glass transition temperature of miscible blends at various polymer mass fractions.

(1) $$\ln\left(T_{g}/T_{g,I}\right)=\frac{w_{II}\Delta c_{p,II}\ln\left(T_{g,II}/T_{g,I}\right)}{w_{I}\Delta c_{p,I}+w_{II}\Delta c_{p,II}}$$

(2) $$\frac{1}{T_{g}}=\frac{w_{I}}{T_{g,I}}+\frac{w_{II}}{T_{g,II}}$$

In Equations (1) and (2), $T_{g}$ is the glass transition temperature of the blend,$\;w_{I}$ and $w_{II}$ are mass fractions,$\;\Delta c_{p,I}$ and $\Delta c_{p,II}$ are the differences of the heat capacity when changing from the glassy to the rubbery state measured using DSC, and $T_{g,I}$ and $T_{g,II}$ are the glass transition temperatures of polymers I and II respectively. 

High pressure differential scanning calorimetry (HP-DSC) measurements were carried out using a calorimeter HP-DSC 1 (Mettler Toledo), and analyzed using STARe SW 16.20 software (Mettler Toledo). The aluminum pan with a multi-perforated lid was filled with approximately 7 mg of powdered polymer. The sample was rinsed with CO_2_ for 5 min in the equipment. First, a heating rate of 10 K min^−1^ was applied from 25 °C to 260 °C in a CO_2_ atmosphere at 1 bar. Then, the sample was allowed to cool down to 150 °C and then held at this temperature for 3 h at the desired CO_2_ pressure (1 bar, 10 bar, 20 bar or 30 bar). The sample was then heated up to 260 °C at a heating rate of 10 K/min while maintaining the CO_2_ pressure. Considering the diffusion coefficient of *D* = 3 × 10^−8^ cm^2^/s for PESU at room temperature [54,55] and the sample particles having radius of *R* = 100–150 µm, the saturation time can be calculated using *t_sat_* = *R*^2^/*D*, i.e., approximately between 55 and 130 min. Since, saturation time decreases with increasing temperature due to softening of the polymer, the selected time of 3 h is sufficient for saturation for PESU and the blends at 150 °C. The *T_g_* was determined by analysing the final heating interval when the sample was assumed to be saturated with CO_2_. 

For rheological measurements, cylindrical samples measuring 8 mm in diameter and 2 mm in thickness were prepared using compression molding at 270 °C for a total time of 10 min. For compression molding of PESU and blend samples, a hot press (Paul-Otto Weber, Remshalden, Germany) was used. As PVP K 30 was available in powder form, cylindrical samples measuring 8 mm in diameter and 2 mm in thickness were prepared using Vacuum MR Hei-End (MeltPrep GmbH, Graz, Austria) at 240 °C. It was ensured that the samples had no air bubbles, cracks, weld lines or rough surfaces through visual inspection. Rheological measurements were carried out on an Anton Paar MCR 502 rheometer with a plate-plate geometry. Frequency sweeps in the frequency range between 0.01 and 100 rad/s were carried out at temperatures 190, 200, 220, 240 and 260 °C for PVP K 30, and at 260, 280, 300 and 320 °C for the other materials. The frequency sweeps started with the highest frequency. The master curves were constructed using the software LSSHIFT developed by Honerkamp and Weese in 1993 [56]. For amorphous polymers, the dependence of the horizontal shift factor $a_{T}$ on temperature T in the construction of master curves can be described using the William-Landel-Ferry (WLF) equation [57],

(3) $$\log\left(a_{T}\right)=-\frac{c_{1}\left(T-T_{ref}\right)}{c_{2}+\left(T-T_{ref}\right)}$$

The WLF parameters $c_{1}$ and $c_{2}$ were obtained by applying a least-squares fit of Equation (2) to the master curves at reference temperature $T_{ref}$ using LSSHIFT. 

### 3.2. Batch Foaming 

The batch foaming process was carried out on samples similar to those used for rheological measurements. Batch foaming can be divided into two stages. In stage one, i.e., the loading phase, samples were placed in a high pressure vessel (highpreactor BHM-500, Berghof, Eningen, Germany). CO_2_ gas was used as a foaming agent and was inserted into the vessel through an inlet from a dip-tube bottle (99.995% purity, Linde PLC, Dublin, Ireland) using a high pressure syringe pump (Teledyne ISCO, Lincoln, NE, USA) at room temperature up to a pressure value that was calculated based on the combined gas law using the desired temperature and CO_2_ pressure. The vessel was then heated to the desired loading temperature ranging from 125 to 175 °C. This temperature was maintained for a loading time of 24 or 48 h, depending on the experiment. In some trials, water was used as a co-foaming agent along with CO_2_. In these trials, the vessel was filled with 40 mL ultrapure water. Immediately after completion of the loading interval, the pressure was released in a controlled manner for 7 s ensuring a pressure drop to ambient pressure and to initiate foaming (stage 2). Immediately, the samples were placed for 100 s between two hot plates of a hot-press at the desired foaming temperature that ranged between 210 to 270 °C. 

### 3.3. Membrane Manufacturing

**Figure 1:**
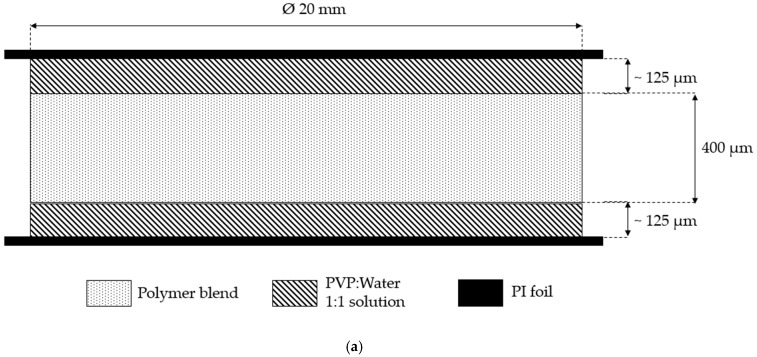

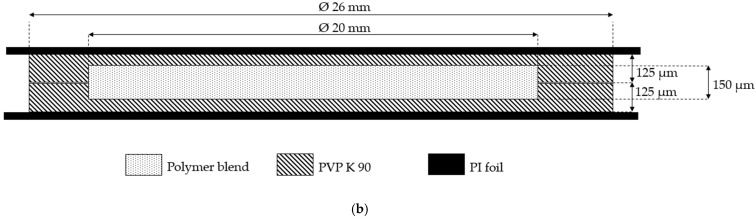
Scheme of a sandwich-type sample: (**a**) Method I; (**b**) Method II.

Blends H-8 and H-32 (with weight fractions 92/08 wt% and 68/32 wt%, respectively) were the main materials of interest for manufacture of membranes and were additionally produced by blending PESU E 3020 P with PVP K 30 in a twin screw extruder (Brabender, Duisburg, Germany). Blend samples for preparing sandwich-type layers were prepared by compression molding. These sandwich-type samples contained a separate PVP layer where BASF Luvitec^®^ K 90 (PVP K 90) was used due to higher ductility and mechanical stability compared to PVP K 30. Each layer was manufactured separately with the dimensions shown in Figure 1. Two manufacturing methods were used, method I (Figure 1a) where PVP layers were made from an aqueous PVP solution (ratio 50/50 wt%), and method II (Figure 1b) where compression molded PVP layers were used. For both methods, the blend films were manufactured using compression molding at 270 °C. In the first method, the blend sample was adhered to the polyimide foil using the aqueous PVP solution as PVP has adhesive properties [58]. The samples were allowed to dry for up to 24 h. In the second method, the temperature used for compression molding was 240 °C for PVP K 90. The layers, as shown in Figure 1b were placed on top of one another and pressed for 3 min together at 205 °C under 20 kN loading using a hot press (Paul-Otto Weber). Polyimide foil was used at the top and the bottom layer as a protection during handling. In both methods, the polymer blend film would thus be completely covered by another polymer, i.e., PVP K 90, and would be implemented as a membrane later. Batch foaming was carried out on these samples using the same process explored previously. The first method of sandwich-type samples was initially implemented on both blends and the second method was only used with the better performing blend.

The foamed samples were subjected to post-treatment using an aqueous solution of 0.1 wt% sodium hypochlorite (pH = 11.5). Samples were initially inserted as a solution in a closed glass bottle at a maintained temperature of 80 °C for 24 h. During this time the solution became saturated with PVP K 90 and the PI foils detached themselves. The polymer of interest with some remenents of PVP K 90 on its surface was carefully transferred in a new solution of NaOCl in a new bottle and subjected to a temperature of 80 °C for 48 h. The choice of this temperature was based on the results of solubility tests of non-foamed polymer films (cf. Supporting Information). To wash out the residual NaOCl and active chlorine, the samples were rinsed in decalcified water at 35 °C for 10 min, aqueous solution of 0.5 wt% sodium bisulfite at 20 °C for 10 min and finally with decalcified water at 80 °C for 10 min [33].

### 3.4. Foam Characterization and Membrane Properties

Scanning electron microscopy (SEM) was used to characterize the foams. For large samples, foams were broken using liquid nitrogen to retain their nanostructure. For smaller samples, foams were cut using a sanitized sharp razor blade which caused smearing of the nanoscale structures present in the cutting plane. The average cell size was measured for selected foams using the scanning electron micrographs and the measurement tool in Photoshop CS6 (Adobe, San Jose, CA, USA). The porosity was measured for selected foams from the micrographs by measuring the number of pixels taken by visible cells and calculating the percentage versus the total number of pixels in the micrographs. 

Density measurement was carried out using the buoyancy method on a density measurement device (Mettler Toledo, Gießen, Germany). Water-flux measurements were carried out on samples after completion of post-treatment using a membrane-holding cell with a diameter of 20 mm and an in-house constructed testing facility. The measurements were carried out with decalcified water at 7 bar pressure. 

Retention tests were carried out using a Millipore cell that held a solution of 0.01 wt% poly(ethylene oxide) (PEO) in water of average molecular weight 400,000 Da. The broad molecular weight distribution of the chosen PEO allowed filterability of various length molecules to be observed. The solution was allowed to mix thoroughly using constant magnetic stirring in a closed flask for 24 h before using in retention tests. The feed solution and the permeate solutions were collected and further analysed using gel permeation chromatography (GPC). The retention coefficient R  was calculated using the Equation (4) where, $w_{P}$ and $w_{F}$ are the mass fractions of PEO in permeate and feed solutions respectively.

(4) $$R=1-\frac{w_{P}}{w_{F}}$$

## 4. Results and Discussion

### 4.1. Material Characterization

**Figure 2:**
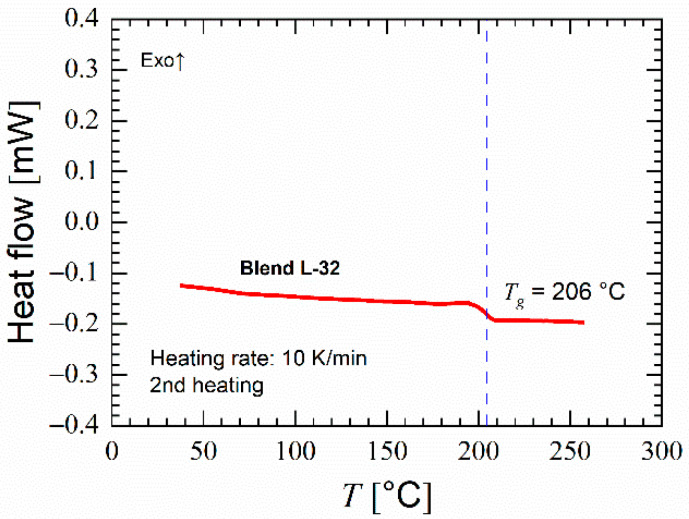
Heat flow versus temperature in second heating of blend L-32 showing single glass transition.

In our DSC measurements, all homopolymers and blends showed a single glass transition. As an example, Figure 2 shows the DSC graph of blend L-32.

**Figure 3:**
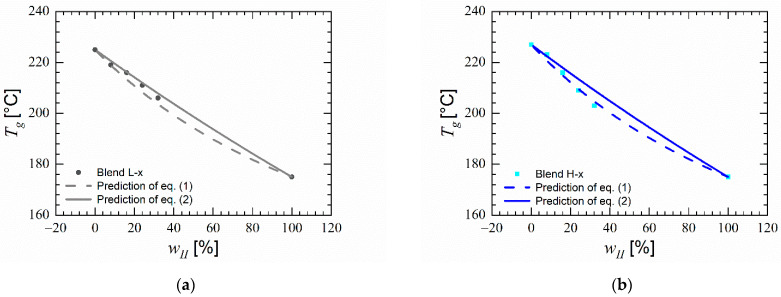
Glass transition temperature versus weight concentration of PVP in blends and predictions of Equations (1) and (2) based on glass transition temperatures of homopolymers; (**a**) Blends with PESU E 2010 (L-x) (**b**) Blends with PESU E 3020 P (H-x).

This provides an indication that the homopolymer chains are mixed on a segmental level in a single phase within these blends. As seen in Figure 3a,b, the blends with high molecular weight PESU undergo a higher reduction of glass transition temperature with increase in PVP content than low molecular weight PESU. This suggests a higher reduction of free volume caused by PVP K 30 in the blends with higher molecular weight PESU than in the blends with lower molecular weight PESU. Figure 3a,b also show the change in glass transition temperature due to change in polymer content as prediction of Equations (1) and (2) based on the glass transition temperatures of polyethersulfone as polymer I and poly(*N*-vinyl pyrrolidone) as polymer II. Blends with PESU E 2010 exibited glass transition temperatures near the predicted values of both equations, whereas in blends with PESU E 3020 P the observed glass transition temperatures are more close to the values predicted by Equation (1). The predictions of Equation (1) correspond closely with both L-x and H-x blend combinations as the change in heat capacities at glass transition are dissimilar for both blend components and the ratio between their glass transition temperatures is unequal to unity. As stated by Couchman [52], when a polymer blend fulfils the premise of Equation (1), it provides an indication that the blend is composed of miscible polymers.

**Figure 4:**
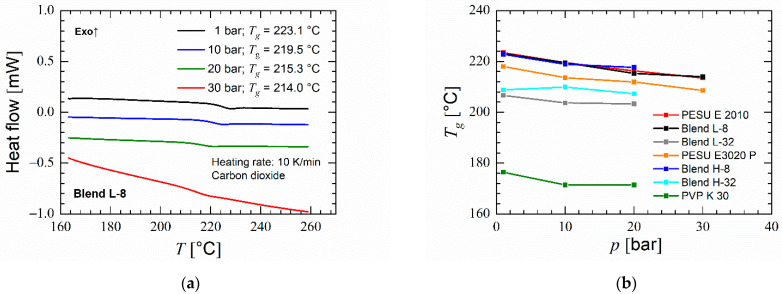
HP-DSC results for selected blends: (**a**) Effect of CO_2_ pressure on glass transition temperature of blend L-8 (**b**) *T_g_* at various CO_2_ pressures (Some materials did not show an identifiable glass transition at 30 bar).

The influence of CO_2_ on the thermal properties of the materials was studied by using HP-DSC. Compared to the sharp glass transition occurring observed by DSC under nitrogen, the glass transition occurs gradually spreading over a larger range of temperature. This spread increases with increasing CO_2_ pressure as seen in Figure 4a for blend L-8. In some blends and materials, the glass transition occurred gradually without a distinct inflection point. Figure 4b shows a linear decrease of glass transition temperature with increasing CO_2_ pressure for both grades of PESU. However, for PVP K 30 the glass transition temperature stayed constant above 10 bar CO_2_ pressure. These influences on PESU and PVP act proportionally in the blends according to their weight contents. With increasing CO_2_ pressure blends with 8% PVP content undergo a larger decrease in *T_g_* than blends with 32% PVP. A larger free volume in a polymer leads to a lower glass transition temperature. Therefore, the HP-DSC measurements show that CO_2_ has a larger effect on the free volume in PESU than PVP K 30 [59,60]. Foaming was observed in some of the samples after removal from the device. 

**Figure 5:**
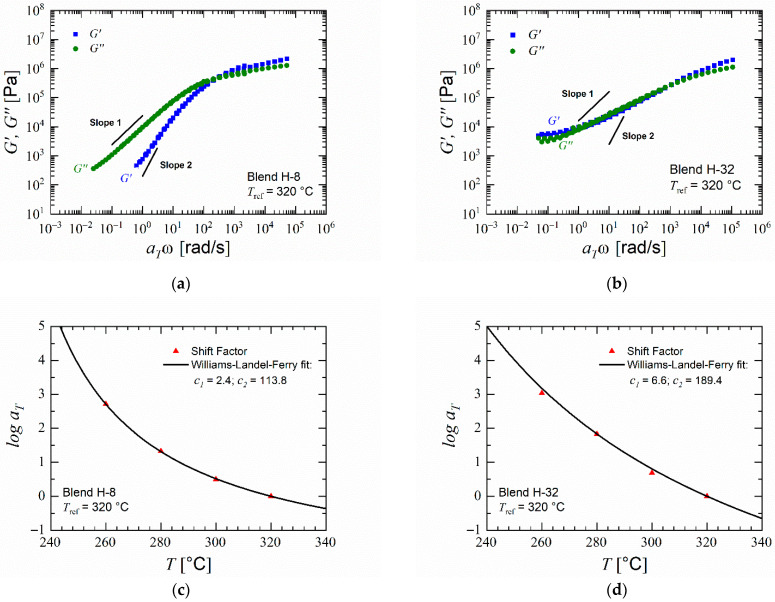
Rheological analysis of selected materials: (**a**,**b**) Master curve of blends H-8 and H-32; (**c**,**d**) Shift factor and WLF fit of blends H-8 and H-32.

The effects of composition and type of the polymer blends on the dynamic moduli of the blends were determined by rheological analysis. In PESU, the storage modulus G′ remains lower than the loss modulus G″ at frequencies lower than the crossover point. With decreasing angular frequency, in double logarithmic presentation, the storage modulus decreases with a slope nearly equal to 2 and the loss modulus with a slope nearly equal to 1. PVP K 30 does not adhere to the slopes equal to one and two of storage and loss modulus, respectively, and shows an increasing storage modulus with decreasing angular frequency because of thermally induced crosslinking. Since crosslinking increases the elasticity of the polymer, it could lead to a limited foam expansion and collapse during foaming of pure PVP or blends with high content of PVP. The data for pristine PESU and PVP are provided in the Supporting Information. The master curves of the moduli vs. angular frequency measurements shown in Figure 5a,b show that the blends translate the behaviors of PESU and PVP K 30 with their polymer contents correspondingly. Since both PESU and PVP are amorphous polymers [61,62], the temperature dependence of the horizontal shift factor in the construction of master curves for their blends can be described using the Equation (3) where reference temperature of 320 °C was chosen. As seen in Figure 5c,d, both blends obey the WLF equation which provides another evidence that PESU and PVP are miscible at both high and low concentrations of PVP [63,64]. 

### 4.2. Batch Foaming

In the experiments where CO_2_ was used as a foaming agent, all selected materials yielded mostly closed-cellular foams. PVP K 30 delivered the lowest density foams among all materials in most trials. However, in some trials, during the foaming stage, the PVP samples initially grew into 3–4 times the original size as observed visually, and collapsed instantaneously after 45 to 60 s of exposure to the foaming temperature. This foam collapse occurred only when the loading pressure was set to 50 bar but did not occur in the trials conducted at 100 bar. As PVP is a highly elastic material in the melt state, the expansion caused by bubble growth is easily reversed, thus attaining the original size. Figure S10 in the Supporting Information show that this collapse has resulted into a crushed foam structure. Foams of blends with 32% PVP had the highest porosity among the blends. This increase in porosity due to increase of PVP content supports the findings by Shi et al. [41] where the increase in the polymer content of the minor component in the single-phase polymer blend yielded in higher porosity. 

**Figure 6:**
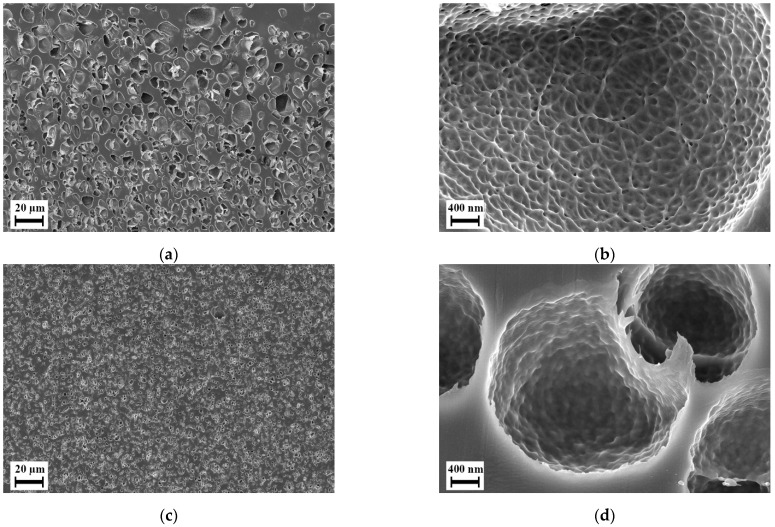
Scanning electron micrographs of selected foams manufactured using foaming agent CO_2_: (**a**,**b**) Blend L-8; (**c**,**d**) Blend H-8.

Although majorly closed microcellular foams, the blends L-8 and H-8 exhibit a certain nanoscale structure on the cell-walls of the microcellular foams as shown in Figure 6. As discussed in the Introduction, many researchers have found this structure in foams of various polymers at certain processing conditions. Fukasawa et al. [65] and Gong et al. [19] found a similar structure in polycarbonate foams under certain processing conditions. Their structure consisted of partially open pores formed within this structure. Fukasawa et al. [65] linked the formation of these pores to the crystallization of polycarbonate resulting in a fibrillary structure that was stretched as a result of bubble growth in the amorphous region during foaming. This explanation was based on various studies that focused on the crystallization of polycarbonate where such fibrillary structure was also reported. However, this explanation remains a speculation as evidence of CO_2_ induced crystallization cannot be identified in their samples. Gong et al. [19] and Guo et al. [21,22,23] provide explanations that seem more plausible towards the cause of this structure. Gong et al. [19] explained that the formation of the fibril structure on the cell walls is due to the phenomenon called crazing. The nucleation and bubble growth induced due to foaming in the polymer results in biaxial tensile deformation. Thus, the fibrils are stretched and result in the formation of voids. They also found a direct relation of the strain energy around the pores during nucleation and bubble growth towards the formation of the nanostructure. Guo et al. [21,22,23] found a similar structure in polysulfone, polyphenylsulfone and a cyclic olefin copolymer. They named the structure as bicontinuous structure and suggest that an open or closed nanoscale structure may be a result of stress induced nucleation. Although the reasoning given by Gong et al. [19] and Guo et al. [21,22,23] differ, they agree in that the structure is caused by stretching induced by the growth of micro cells. 

In our case, the blends L-8 and H-8 delivered similar structure on the cell-walls of the microcells, which however is not open porous. This can be explained by the lack of sufficient deformation provided during the growth of microcells since a higher expansion during foaming is required for fabrication of open-celled foams [22,23]. A higher expansion would lead towards streching the nanostructure enough such that open nanocellular foams are obtained.

Initially in the trials with CO_2_ and water, the effect of foaming temperature was analyzed. The parameters loading temperature (150 °C), loading time (48 h), pressure (100 bar) and foaming time (100 s) were kept constant.

**Figure 7:**
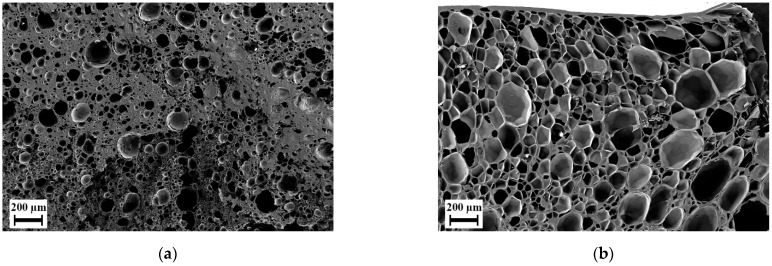
Scanning electron micrographs of foams of blend H-32 manufactured at loading time 48 h, pressure 100 bar, loading temperature 150 °C, foaming time 100 s and foaming temperature 230 °C: (**a**) Using only CO_2_ as foaming agent; (**b**) Using CO_2_ and H_2_O as foaming agents.

**Figure 8:**
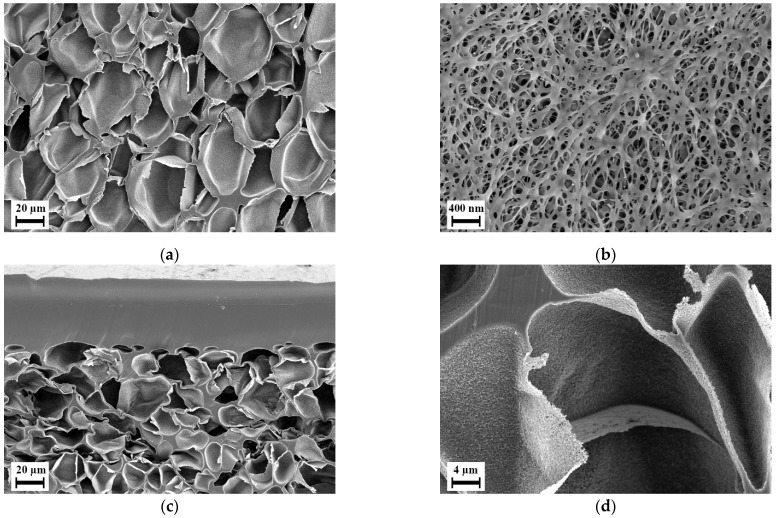
Scanning electron micrographs of foams manufactured using blowing agent CO_2_ and H_2_O at loading time 48 h, pressure 100 bar, loading temperature 150 °C, foaming time 100 s and foaming temperature 230 °C for Blend H-8: (**a**) internal structure at 500× magnification; (**b**) internal structure at 20,000× magnification; (**c**) structure near the surface at 500× magnification; (**d**) internal structure at 2500× magnification.

**Figure 9:**
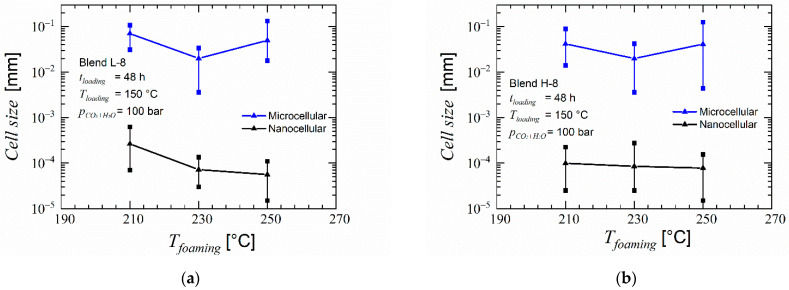

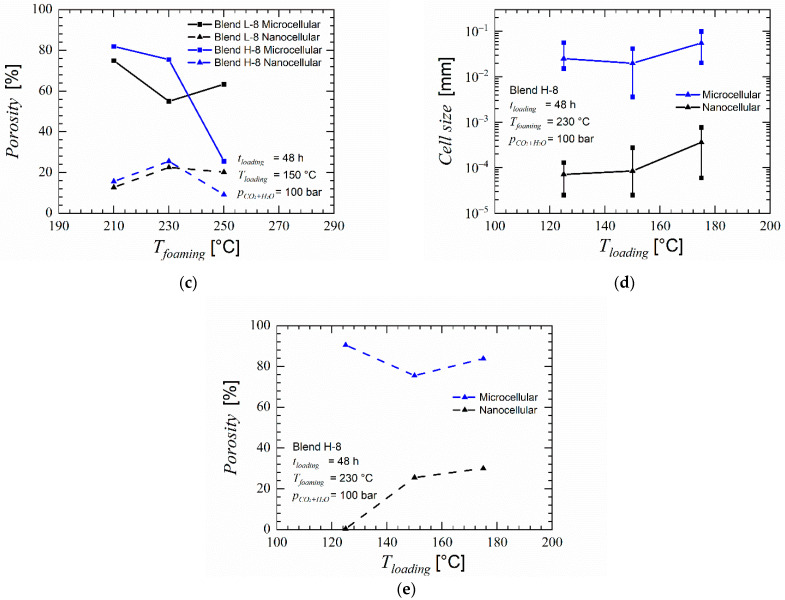
Average cell size and porosity: (**a**) Average cell size vs. foaming temperature in foams of blend L-8; (**b**) Average cell size vs. foaming temperature in foams of blend H-8; (**c**) Porosity vs. foaming temperature in foams of blends L-8 and H-8; (**d**) Average cell size vs. loading temperature in foams of blend H-8; (**e**) Porosity vs. loading temperature in foams of blend H-8.

As expected, when using CO_2_ and water as foaming agent an increased porosity was found in foams of blends H-8 and H-32 in the microcells as seen in Figure 7 and Figure 8. Blend H-32 showed a higher porosity and partially open-cellular foam, but failed in providing any nanoscale structure. Foams of blends L-8 (cf. Supporting Information Figure S7) and H-8 contained high uniformity and similar cell size. The walls of these cells were made of a mesh of open pores smaller than 200 nm. The increased porosity enabled formation of pores within the nanoscale structure. This structure was seen at samples foamed at temperatures of 210, 230 and 250 °C for both materials. At 270 °C, this structure was not found. Comparing the porosity and the cell size distribution, the foaming temperature 230 °C provides an average cell size below 100 nm and the highest porosity of this type of pores was larger than 20%. At this foaming temperature, among the two blends, H-8 blend provides the lowest average cell size while maintaining a high porosity in both microcells and the smaller open pores. To maintain connectivity between the microcells through the smaller open pores, an overall high porosity is also desired. Therefore, blend H-8 is taken as an optimum candidate for the further tests by selecting the foaming temperature as 230 °C. As shown in Figure 9, the average size of the pores on the microcell walls in the foams obtained using loading temperature as 150 °C is below 100 nm and has a porosity larger than 25%. The loading temperature 175 °C delivers higher porosity and larger cell size than the loading temperature 150 °C in both microcells and the pores on their cell-walls. Scanning electron micrographs of the effect of loading temperature is available in the Supporting Information as Figure S9. A higher loading temperature softens the polymer more and aids in faster diffusion of the foaming agents. This results in higher porosity and larger microcells during the foaming stage. An increased growth of microcells causes higher stretching of the cell walls wherein the nodal structures aid in creation of larger pores. Similarly, a lower loading temperature causes a lower solubility of foaming agents in the polymer and results in low porosity and smaller cell sizes. Therefore, the originally selected loading temperature of 150 °C appears ideal in this case for the blend H-8 such that an interconnectivity is achieved. 

Owusu-Nkwantabisah et al. [29] analysed the effects of using H_2_O along with CO_2_ as a foaming agent for foaming of polyethersulfone and found that using scCO_2_ + shH_2_O as foaming agent delivered an increased porosity and in some cases, open pores. As the structure found in foams H-8 and L-8 previously could develop open pores if the overall porosity is increased, scCO_2_ + shH_2_O was used as foaming agent.

### 4.3. Membranes

All foams discussed in Section 3.2, exhibit a solid non-foamed skin layer of approximately 20–50 µm thickness similar to as shown in Figure 9c. To use these foams for their permeation properties, this outer layer is necessary to be eliminated and thus sandwich-type samples were used. The sandwich-type samples have an additional layer of another polymer, PVP K 90 in this case, such that it behaves as a faux outer layer where the escape of diffused foaming agent would take place during depressurization thus limiting the non-foamed surface layer towards the PVP K 90. This allows the polymer of interest to stay completely foamed. During post-treatment, the PVP layers dissolve into the NaOCl solution and the thoroughly foamed polymer of interest is obtained. 

**Figure 10:**
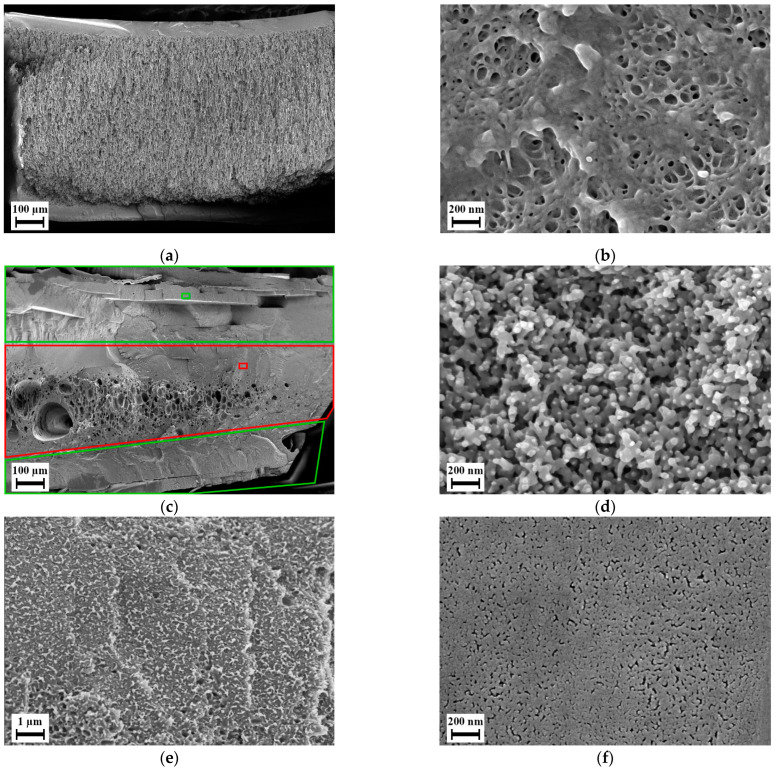
Scanning electron micrographs of sandwich-type samples manufactured using method I, batch foamed and post-treated: (**a**) Cross section of blend H-8; (**b**) Microstructure observed in the skin layer from figure (**a**); (**c**) Cross section of blend H-32; (**d**) microstructure observed in smaller green box and commonly found across the area enclosed by larger green boxes in figure (**c**); (**e**) Observed solid structure in the smaller red box and commonly found across the area enclosed by the larger red box in figure (**c**); (**f**) Outer surface of blend H-32.

During the batch foaming of sandwich-type samples that were prepared using aqueous solution of PVP, blend H-8 showed a similar internal foam structure as it was seen in the previous batch foaming experiments. However, the surface layer was foamed with a separate foaming pattern, i.e., with smaller closed-cells with an average diameter of 100 nm and blind open pores on the surface. Figure 10a,b show the microstructure of foam of blend H-8 manufactured using sandwich method. The influence of using the sandwich-type samples can be seen but applicable results were not delivered. Blend H-32 however, yielded a completely permeable open nanocellular foam with slit type open pores as shown in Figure 10d on the surface with average width 50 nm as shown in Figure 10f. This foam structure was available only in the regions indicated in green in Figure 10c. The region highlighted in red were non-foamed as seen in Figure 10e. This indicated that the blend layer was too thick. The sample could therefore not be used for testing permeation. Also, in some samples, the blend layer was not fully covered by the PVP due to the adhesive solution not flowing in certain areas between the PI foil and the blend. This caused certain samples to have non-foamed skin layers in the regions where the blend film was not covered by PVP K 90. 

**Figure 11:**
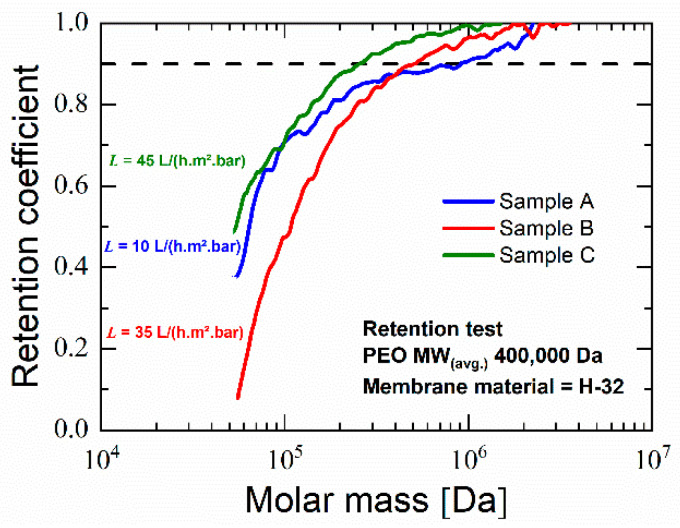
Retention coefficient of membrane manufactured using sandwich-type samples vs. molar mass.

**Figure 12:**
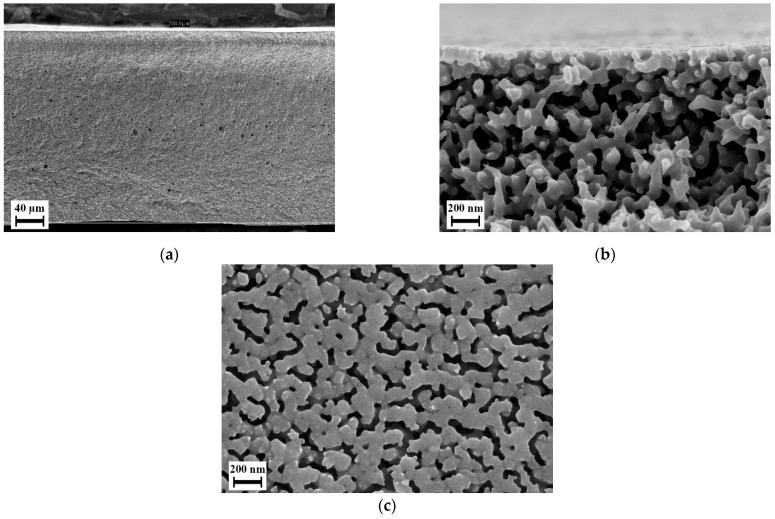
Scanning electron micrographs of sandwich-type sample manufactured using method II with blend H-32, after batch foaming and post-treatment: (**a**) cross section; (**b**) internal structure; (**c**) surface.

To overcome these issues, thinner blend samples were used in method II of preparing sandwich-type samples, and to ensure completely sealed covering of the blend layer, the PVP layer was compression molded instead. Since only blend H-32 delivered promising results, only blend H-32 was subjected to further experiments using the second method of preparing sandwich-type samples. After batch foaming, along with the blend layer, the outer PVP K 90 layer foamed. This PVP foam could be dissolved during post-treatment. Figure 11 show that the blend layer yields a completely permeable open-cellular foam with cell sizes less than 300 nm. The membranes after post-treatment as shown in Figure 12 had a thickness in the range of 300 to 350 µm, approximately two times thicker than the polymer layer in the sandwich-type samples. This open-cellular structure was not observed in the foams of blend H-32 that were manufactured during the batch foaming trials using cylindrical samples as seen in Figure 7. Comparison of the morphologies after the loading phase of batch foaming, after foaming and after post-treatment, shows that the internal open-cellular structure is obtained only after foaming and the post-treatment aids in the removal of the outer PVP layer and provides an open porous surface. This open porous surface has an average cell size around 50 nm and is slightly larger than the internal porous structure of the foam which is around 200 nm. Therefore, due to this pore size, this porous surface is capable of functioning as a selective layer for ultrafiltration [2]. 

**Figure 13:**
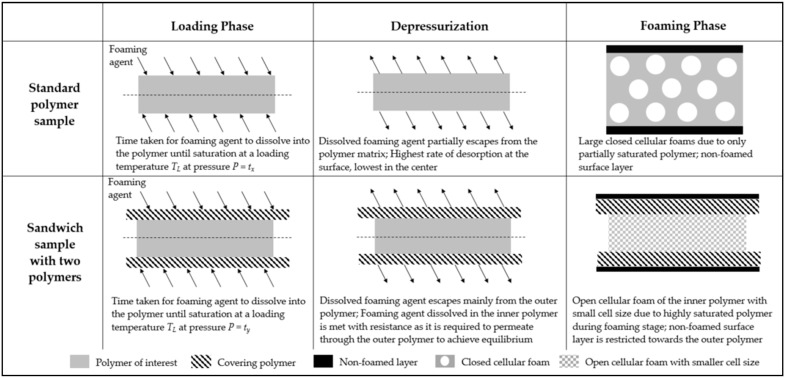
Functioning of sandwich-type sample during batch foaming versus a standard sample.

**Figure 14:**
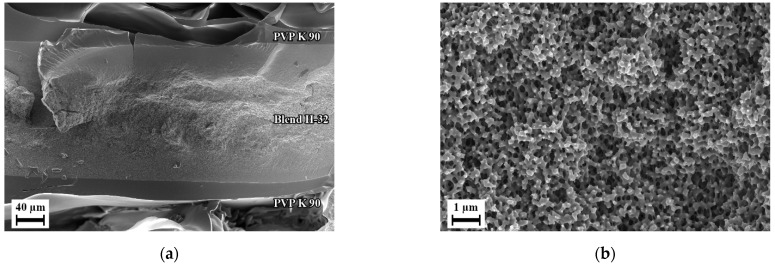
Scanning electron micrographs of sandwich-type sample manufactured using method II with blend H-32 after batch foaming before post-treatment: (**a**) at 200× magnification; (**b**) at 10,000× magnification on layer with Blend H-32.

The mechanism behind the realization of such a structure using sandwich-type samples is depicted in Figure 13. In a standard sample, during the loading phase, the foaming agent dissolves within the sample over time *t_x_* and attains near-saturated state. During depressurization of the batch foaming reactor, the dissolved foaming agent begins to escape from the polymer matrix. The rate of escaping is highest at the surface and lowest in the middle of the sample. This occurs as long as the depressurization time along with the time taken to transfer the samples from the reactor to the foaming temperatures, is larger than zero. Therefore, the samples are no more in their fully saturated state. This yields low level of nucleation and causes closed-cellular foams with non-foamed outer surface layer. Therefore, blend H-32 when foamed as a standard sample, yielded closed microcellular foam as seen in Figure 7b. In sandwich-type samples, when one polymer is covered by another polymer, under similar conditions the amount of time taken for the foaming agent to dissolve within both polymers until saturation, *t_y_* is larger than time *t_x_* due to larger thickness and different diffusion coefficients. During depressurization, similar to standard samples, the foaming agent begins escaping from the sample, but this occurs mainly in the outer polymer. As the internal polymer layer is tightly enclosed by the outer polymer layer, any escape of foaming agent from internal polymer would need permeation of the foaming agent through the outer polymer layer. As the outer polymer is different from the inner polymer, they have different permeation and solubility properties. The internal polymer remains near to its completely saturated state and delivers highly porous nanocellular foam whereas, the non-foamed outer surface layer is restricted to the outer polymer. Therefore, based on this principle, an open-celled foam with cell size in nanometers was realized in blend H-32 using this method as seen in Figure 14. Removal of the outer polymer was required to use the inner polymer for any applications. Thus, selection of the outer polymer as a water-soluble or inorganic solvent soluble polymer is essential such that the post-treatment of the sandwich-type sample would lead to dissolution of the outer layer and the internal layer stays unaffected. 

Three membrane samples from blend H-32, viz. A, B and C were tested for water flux and yielded a flux of 10, 35 and 45 L/(h m^2^ bar), respectively. These values of water flux are similar to those seen with polyethersulfone ultrafiltration membranes manufactured using methods involving organic solvent [66,67]. Since larger membrane thickness adds further resistance to water flow, future studies could employ thinner samples so as to increase the water flux. As seen in Figure 11, retention tests reveal a retention coefficient above 0.9 for PEO with molecular weight higher than ~260,000 Da for sample C and an average of ~480,000 Da for all measured samples. Since retention of molecules between 10³–10^6^ Da is classified under ultrafiltration [1,68,69], these values although near the upper limit, provide evidence that these membranes have potential for ultrafiltration. Although the performance of this developed membrane is lower than modern ultrafiltration membranes manufactured using organic solvents [39,70,71,72,73], these membranes provide a genesis towards large-scale development of ultrafiltration membranes using foaming. Future studies focus on optimizations and improvements of the membranes to match the ultrafiltration standards of membranes manufactured using other methods and also on developing ways to manufacture them efficiently and on a large scale. 

## 5. Conclusions

Batch foaming experiments on PESU/PVP blends using CO_2_ as blowing agent show a clear influence of PVP content on the foaming behavior. Foams of PESU blends with 32% PVP exhibited the highest density and foams of blends with 8% PVP show a tendency towards formation of a fibril structure on the cell walls of the closed-cells. Using superheated H_2_O with CO_2_ as foaming agents the porosity of the foams was significantly increased. This lead towards increased expansion of the fibril structures on the walls of foam cells thus creating a smaller porous structure connecting the larger cells. Optimal processing conditions and blend compositions for obtaining high porosity and lowest cell size were found. The samples, however, contain a solid non-foamed surface layer which hinders the direct application of the open-celled foam as membranes. Sample preparation methods developed by us eliminated the formation of the non-foamed surface layer, and at the same time allowed the sample to be in near-saturated state. Using this method, blend with 8% PVP foams completely including the outer surface but cannot deliver a complete open-cellular foam. A blend with 32% PVP however, due to high saturation of foaming agent, exhibits complete open-cellular foam containing porous surface with average surface cell size of 50 nm and average internal cell size of 200 nm. These open nanocellular foams provide an average water flux of 30 L/(h m^2^ bar) and an average retention coefficient above 0.9 for PEO with molecular weights above 480,000 Da confirming the proof of concept for proposed application of these foams as ultrafiltration membranes. 

## Figures and Tables

**Table 1:** Nomenclature of blends with respect to their composition.

Blend Name	PESU		PVP K 30
	Type	Content [wt%]	Content [wt%]
L-8	PESU E 2010	92	8
L-16	PESU E 2010	84	16
L-24	PESU E 2010	76	24
L-32	PESU E 2010	68	32
H-8	PESU E 3020 P	92	8
H-16	PESU E 3020 P	84	16
H-24	PESU E 3020 P	76	24
H-32	PESU E 3020 P	68	32

## Data Availability

The characterization data are available upon request from the authors.

## Supplementary Materials

The following are available online at https://www.mdpi.com/article/10.3390/polym14061177/s1, Figure S1: Time taken to dissolve PVP K 30 samples in water at various temperatures. Figure S2: Scanning electron micrographs of post-treated blends in NaOCl at various temperatures: (a) Surface of film of blend H-8 at 80 °C (b) Surface of film of blend H-32 at RT; (c) Surface of film of blend H-32 at 80 °C; (d) Cross-section of film of blend H-32 at 80 °C. Figure S3: IR spectra of blend, non-foamed blend H-8 and after foaming with various foaming agents. Figure S4: DSC 2nd heating curves of non-foamed blend H-8 and after foaming with various foaming agents. Figure S5. Master curves of selected materials from rheological analysis: (a) PESU E 2010; (b) PESU E 3020 P; (c) PVP K 30; (d) Blend L-8; (e) Blend L-32. Figure S6. Results of thermogravimetric analysis for selected materials. Figure S7. Scanning electron micrographs of blend L-8 foams manufactured using blowing agents CO_2_ and H_2_O at loading time 48 h, pressure 100 bar, loading temperature 150 °C, foaming time 100 s and various foaming temperatures: (a,b) 210 °C; (c,d) 230 °C; (e,f) 250 °C; (g,h) 270 °C. Figure S8. Scanning electron micrographs of blend H-8 foams manufactured using blowing agents CO_2_ and H_2_O at loading time 48 h, pressure 100 bar, loading temperature 150 °C, foaming time 100 s and various foaming temperatures: (a,b) 210 °C; (c,d) 250 °C; (e,f) 270 °C. Figure S9. Scanning electron micrographs of foams of blend H-8 at the loading time 48 h, pressure 100 bar, foaming temperature 230 °C, foaming time 100 s, the blowing agent CO_2_ and H_2_O and various loading temperatures: (a,b): 125 °C; (c,d): 175 °C. Figure S10. Scanning electron micrograph of collapsed PVP foam manufactured using blowing agent CO_2_ at loading time 48 h, pressure 50 bar, loading temperature 150 °C, foaming time 100 s and foaming temperature 250 °C. Figure S11. Scanning electron micrographs of sandwich-type sample manufactured using method II with blend H-32: (a,b) after loading phase without subjecting to foaming temperatures (Similar settings and method used as Figure 12).

## Abbreviation

Symbols used in Equations (1)–(3).

**Symbol**	**Parameter**	**Unit**
*T_g_*	Glass transition temperature of the blend	K
*w* *_I_*	Mass fraction of polymer I	-
*c_p,_* *_I_*	Heat capacity of polymer I	mW
*T_g,_* *_I_*	Glass transition temperature of polymer I	K
*W* *_II_*	Mass fraction of polymer II	-
*c_p,_* *_II_*	Heat capacity of polymer II	mW
*T_g_* *_,II_*	Glass transition temperature of polymer II	K
$a_{T}$	Horizontal shift factor	-
$c_{1},\;c_{2}$	WLF parameters	K
T	Temperature	°C
$T_{ref}$	Reference temperature	°C
R	Retention coefficient	-
$w_{P}$	Mass fraction of PEO in permeate	-
$w_{F}$	Mass fraction of PEO in feed	-

## References

[1] Zhao Y., Youcai Z. (2018). Chapter 2—Physical and chemical treatment processes for leachate. Pollution Control Technology for Leachate from Municipal Solid Waste.

[2] Singh R. (2015). Chapter 1—Introduction to Membrane Technology. Membrane Technology and Engineering for Water Purification.

[3] Zhang F. (2009). Preparation of cation exchange resin filled EVAL hollow fiber membrane adsorbent. Int. J. Chem..

[4] Verissimo S., Peinemann K.V., Bordado J. (2005). Thin-film composite hollow fiber membranes: An optimized manufacturing method. J. Membr. Sci..

[5] Wang D.L., Teo W.K., Li K. (2002). Preparation and characterization of high-flux polysulfone hollow fibre gas separation membranes. J. Membr. Sci..

[6] Bulte A.M.W., Mulder M.H.V., Smolders C.A., Strathmann H. (1996). Diffusion induced phase separation with crystallizable nylons. 1. Mass transfer processes for nylon 4,6. J. Membr. Sci..

[7] Bulte A.M.W., Mulder M.H.V., Smolders C.A., Strathmann H. (1996). Diffusion induced phase separation with crystallizable nylons. 2. Relation to final membrane morphology. J. Membr. Sci..

[8] Preventing Adverse Health Effects from Exposure to: Dimethylformamide (DMF). https://www.cdc.gov/niosh/docs/90-105/default.html.

[9] Åkesson D.B. N-Methyl-2-pyrrolidone. https://www.who.int/ipcs/publications/cicad/en/cicad35.pdf.

[10] Dimethyl Acetamide. https://nj.gov/health/eoh/rtkweb/documents/fs/0736.pdf.

[11] ECHA E.C.A. Formic Acid. https://echa.europa.eu/de/substance-information/-/substanceinfo/100.000.527.

[12] Redlich C.A., Beckett W.S., Sparer J., Barwick K.W., Riely C.A., Miller H., Sigal S.L., Shalat S.L., Cullen M.R. (1988). Liver-disease associated with occupational exposure to the solvent dimethylformamide. Ann. Intern. Med..

[13] Rundquist E.M., Pink C.J., Livingston A.G. (2012). Organic solvent nanofiltration: A potential alternative to distillation for solvent recovery from crystallisation mother liquors. Green Chem..

[14] Perry R.H., Green D.W., Maloney J.O. (1997). Distillation. Perry’s Chemical Engineers’ Handbook.

[15] Gadalla M.A., Olujic Z., Jansens P.J., Jobson M., Smith R. (2005). Reducing CO_2_ emissions and energy consumption of heat-integrated distillation systems. Environ. Sci. Technol..

[16] Okolieocha C., Raps D., Subramaniam K., Altstädt V. (2015). Microcellular to nanocellular polymer foams: Progress (2004–2015) and future directions—A review. Eur. Polym. J..

[17] Krause B., Boerrigter M.E., van der Vegt N.F.A., Strathmann H., Wessling M. (2001). Novel open-cellular polysulfone morphologies produced with trace concentrations of solvents as pore opener. J. Membr. Sci..

[18] Krause B., van der Vegt N.F.A., Wessling M. (2002). New ways to produce porous polymeric membranes by carbon dioxide foaming. Desalination.

[19] Gong P.J., Taniguchi T., Ohshima M. (2014). Nanoporous structure of the cell walls of polycarbonate foams. J. Mater. Sci..

[20] Sorrentino L., Aurilia M., Iannace S. (2011). Polymeric Foams from High-Performance Thermoplastics. Adv. Polym. Technol..

[21] Guo H.M., Nicolae A., Kumar V. (2015). Solid-state microcellular and nanocellular polysulfone foams. J. Polym. Sci. Pol. Phys..

[22] Guo H.M., Nicolae A., Kumar V. (2016). Fabrication of high temperature polyphenylsulfone nanofoams using high pressure liquid carbon dioxide. Cell Polym..

[23] Guo H.M., Kumar V. (2015). Effect of glass transition temperature and saturation temperature on the solid-state microcellular foaming of cyclic olefin copolymer. J. Appl. Polym. Sci..

[24] Li Z.K., Jia Y.B., Bai S.B. (2018). Polysulfone foam with high expansion ratio prepared by supercritical carbon dioxide assisted molding foaming method. RSC Adv..

[25] Hwang Y.D., Cha S.W. (2002). The relationship between gas absorption and the glass transition temperature in a batch microcellular foaming process. Polym. Test..

[26] Sauceau M., Fages J., Common A., Nikitine C., Rodier E. (2011). New challenges in polymer foaming: A review of extrusion processes assisted by supercritical carbon dioxide. Prog. Polym. Sci..

[27] Jacobs L.J.M. (2008). Carbon Dioxide as a Sustainable Means to Control Polymer Foam Morphology. Ph.D. Thesis.

[28] Hu D.D., Gu Y., Liu T., Zhao L. (2018). Microcellular foaming of polysulfones in supercritical CO_2_ and the effect of co-blowing agent. J. Supercrit. Fluid.

[29] Owusu-Nkwantabisah S., Staudt C., Lesser A.J. (2018). Synergy of supercritical CO_2_ and superheated H_2_O for enhanced processability of polyethersulfone towards open cell foams. Polym. Eng. Sci..

[30] Schulze M., Handge U.A., Abetz V. (2017). Preparation and characterisation of open-celled foams using polystyrene-*b*-poly(4-vinylpyridine) and poly(4-methylstyrene)-*b*-poly(4-vinylpyridine) diblock copolymers. Polymer.

[31] Smith R.M. (2002). Extractions with superheated water. J. Chromatogr. A.

[32] Tsehaye M.T., Velizarov S., Van der Bruggen B. (2018). Stability of polyethersulfone membranes to oxidative agents: A review. Polym. Degrad. Stabil..

[33] Grunig L., Handge U.A., Koll J., Gronwald O., Weber M., Hankiewicz B., Scharnagl N., Abetz V. (2020). Hydrophilic dual layer hollow fiber membranes for ultrafiltration. Membranes.

[34] Jaleh B., Zare E., Azizian S., Qanati O., Nasrollahzadeh M., Varma R.S. (2020). Preparation and Characterization of Polyvinylpyrrolidone/Polysulfone Ultrafiltration Membrane Modified by Graphene Oxide and Titanium Dioxide for Enhancing Hydrophilicity and Antifouling Properties. J. Inorg. Organomet. Polym. Mater..

[35] Dibrov G., Kagramanov G., Sudin V., Grushevenko E., Yushkin A., Volkov A. (2020). Influence of sodium hypochlorite treatment on pore size distribution of polysulfone/polyvinylpyrrolidone membranes. Membranes.

[36] Al Malek S.A., Abu Seman M.N., Johnson D., Hilal N. (2012). Formation and characterization of polyethersulfone membranes using different concentrations of polyvinylpyrrolidone. Desalination.

[37] Zhang J., Chen S., Bai H.J., Lu S.F., Xiang Y., Jiang S.P. (2021). Effects of phosphotungstic acid on performance of phosphoric acid doped polyethersulfone-polyvinylpyrrolidone membranes for high temperature fuel cells. Int. J. Hydrogen Energy.

[38] Dai Y., Wang J., Tao P.P., He R.H. (2019). Various hydrophilic carbon dots doped high temperature proton exchange composite membranes based on polyvinylpyrrolidone and polyethersulfone. J. Colloid Interf. Sci..

[39] Gronwald O., Weber M. (2020). AGNIQUE AMD 3L as green solvent for polyethersulfone ultrafiltration membrane preparation. J. Appl. Polym. Sci..

[40] Aili D., Kraglund M.R., Tavacoli J., Chatzichristodoulou C., Jensen J.O. (2020). Polysulfone-polyvinylpyrrolidone blend membranes as electrolytes in alkaline water electrolysis. J. Membr. Sci..

[41] Shi Z.L., Ma X.W., Zhao G.Q., Wang G.L., Zhang L., Li B. (2020). Fabrication of high porosity Nanocellular polymer foams based on PMMA/PVDF blends. Mater. Des..

[42] Costeux S., Khan I., Bunker S.P., Jeon H.K. (2015). Experimental study and modeling of nanofoams formation from single phase acrylic copolymers. J. Cell. Plast..

[43] Sun H.L., Sur G.S., Mark J.E. (2002). Microcellular foams from polyethersulfone and polyphenylsulfone—Preparation and mechanical properties. Eur. Polym. J..

[44] Xie Y.P., Ye F., Chen W.H., Tang J.H., Liu P.J. (2020). Preparation of high-strength and lightweight microcellular polysulfone foam with a segregated CNT network for excellent electromagnetic shielding. RSC Adv..

[45] Abbasi H., Antunes M., Velasco J.I. (2020). Effects of graphene nanoplatelets and cellular structure on the thermal conductivity of polysulfone nanocomposite foams. Polymers.

[46] Antunes M., Abbasi H., Velasco J.I. (2021). The effect of microcellular structure on the dynamic mechanical thermal properties of high-performance nanocomposite foams made of graphene nanoplatelets-filled polysulfone. Polymers.

[47] Abbasi H., Antunes M., Velasco J.I. (2020). Electrical conduction behavior of high-performance microcellular nanocomposites made of graphene nanoplatelet-filled polysulfone. Nanomaterials.

[48] Lu Y.Q., Li S.Y., Chen F.Y., Ma H., Gao C.J., Xue L.X. (2022). Development of coin-shaped ZIF-7 functionalized superhydrophobic polysulfone composite foams for continuous removal of oily contaminants from water. J. Hazard. Mater..

[49] Liu S.Q., Yin S., Duvigneau J., Vancso G.J. (2020). Bubble seeding nanocavities: Multiple polymer foam cell nucleation by polydimethylsiloxane-grafted designer silica nanoparticles. ACS Nano.

[50] Ismail A.F., Mustaffar M.I., Illias R.M., Abdullah M.S. (2006). Effect of dope extrusion rate on morphology and performance of hollow fibers membrane for ultrafiltration. Sep. Purif. Technol..

[51] Jim J.S., Chang C.W., Zhu J., Wu H., Zhang Z.T. (2015). Solubility of poly(vinylpyrrolidone) with different molecular weights in supercritical carbon dioxide. J. Chem. Eng. Data.

[52] Couchman P.R. (1978). Compositional variation of glass-transition temperatures. 2. Application of thermodynamic theory to compatible polymer blends. Macromolecules.

[53] Fox T.G. (1956). Influence of diluent and of copolymer composition on the glass temperature of a polymer system. Bull. Am. Phys. Soc..

[54] Kargar M., Handge U.A. (2021). Numerical simulations of gas sorption experiments in polymers: Influence of aspect ratio and pressure increase rate on the determination of diffusion coefficient. Macromol. Theory Simul..

[55] Sun H.L., Mark E.J. (2002). Preparation, characterization, and mechanical properties of some microcellular polysulfone foams. J. Appl. Polym. Sci..

[56] Honerkamp J., Weese J. (1993). A note on estimating mastercurves. Rheol. Acta.

[57] Ferry J.D. (1980). Viscoelastic Properties of Polymers.

[58] Lee J. (2005). Intrinsic adhesion properties of poly(vinyl pyrrolidone) to pharmaceutical materials: Humidity effect. Macromol. Biosci..

[59] Maeda Y., Paul D.R. (1987). Effect of antiplasticization on gas sorption and transport. 1. polysulfone. J. Polym. Sci. Pol. Phys..

[60] Maeda Y., Paul D.R. (1987). Effect of antiplasticization on gas sorption and transport. 3. free-volume interpretation. J. Polym. Sci. Pol. Phys..

[61] Hiremath P., Nuguru K., Agrahari V., Narang A.S., Badawy S.I.F. (2019). Chapter 8—Material attributes and their impact on wet granulation process performance. Handbook of Pharmaceutical Wet Granulation.

[62] McKeen L.W., McKeen L.W. (2006). 4-Binders. Fluorinated Coatings and Finishes Handbook.

[63] Haenelt T.G., Georgopanos P., Abetz C., Rangou S., Alisch D., Meyer A., Handge U.A., Abetz V. (2014). Morphology and elasticity of polystyrene-block-polyisoprene diblock copolymers in the melt. Korea-Aust. Rheol. J..

[64] Georgopanos P., Rangou S., Haenelt T.G., Abetz C., Meyer A., Filiz V., Handge U.A., Abetz V. (2014). Analysis of glass transition and relaxation processes of low molecular weight polystyrene-b-polyisoprene diblock copolymers. Colloid Polym. Sci..

[65] Fukasawa Y., Chen J., Saito H. (2008). A novel nanoporous structure on the surface of bubbles in polycarbonate foams. J. Polym. Sci. Pol. Phys..

[66] Hliavitskaya T., Plisko T., Bildyukevich A., Lipnizki F., Rodrigues G., Sjolin M. (2020). Modification of PES ultrafiltration membranes by cationic polyelectrolyte Praestol 859: Characterization, performance and application for purification of hemicellulose. Chem. Eng. Res. Des..

[67] Kim D., Vovusha H., Schwingenschlogl U., Nunes S.P. (2017). Polyethersulfone flat sheet and hollow fiber membranes from solutions in ionic liquids. J. Membr. Sci..

[68] Fang X.F., Li J.S., Li X., Pan S.L., Zhang X., Sun X.Y., Shen J.Y., Han W.Q., Wang L.J. (2017). Internal pore decoration with polydopamine nanoparticle on polymeric ultrafiltration membrane for enhanced heavy metal removal. Chem. Eng. J..

[69] Lee E.K., Koros W.J., Meyers R.A. (2003). Membranes, Synthetic, Applications. Encyclopedia of Physical Science and Technology.

[70] Adamczak M., Kaminska G., Bohdziewicz J. (2021). Relationship between the addition of carbon nanotubes and cut-off of ultrafiltration membranes and their effect on retention of microcontaminants. Desalination Water Treat..

[71] Mantel T., Benne P., Parsin S., Ernst M. (2018). Electro-conductive composite gold-polyethersulfone-ultrafiltration-membrane: Characterization of membrane and natural organic matter (NOM) filtration performance at different in-situ applied surface potentials. Membranes.

[72] Fang X.F., Li J.S., Li X., Sun X.Y., Shen J.Y., Han W.Q., Wang L.J. (2015). Polyethyleneimine, an effective additive for polyethersulfone ultrafiltration membrane with enhanced permeability and selectivity. J. Membr. Sci..

[73] Gronwald O., Frost I., Ulbricht M., Shalmani A.K., Panglisch S., Grunig L., Handge U.A., Abetz V., Heijnen M., Weber M. (2020). Hydrophilic poly(phenylene sulfone) membranes for ultrafiltration. Sep. Purif. Technol..

