# Supplementary material for: Open-Celled Foams of Polyethersulfone/Poly(N-vinylpyrrolidone) Blends for Ultrafiltration Applications
Source: Polymers (Basel). 2022 Mar 15;14(6):1177. doi: 10.3390/polym14061177 (PMC8953762; doi:10.3390/polym14061177)
Supplement: Supplementary file 1 [file polymers-14-01177-s001.zip › polymers-1617966-supplementary.pdf]

polymers

Article

Open-Celled Foams of Polyethersulfone/Poly(*N*-vinylpyrrolidone) Blends for Ultrafiltration Applications

Aniket Raje [1], Kristian Buhr [1], Joachim Koll [1], Jelena Lillepärg [1], Volker Abetz [1,2] and Ulrich A. Handge [1,3]*

[1] Institute of Membrane Research, Helmholtz-Zentrum Hereon, Max-Planck-Strasse 1, 21502 Geesthacht, Germany; aniket.raje@hereon.de (A.R.); kristian.buhr@hereon.de (K.B.); joachim.koll@hereon.de (J.K.); jelena.lillepaerg@hereon.de (J.L.); volker.abetz@hereon.de (V.A.)
[2] Institute of Physical Chemistry, Universität Hamburg, Grindelallee 117, 20146 Hamburg, Germany
[3] Chair of Plastics Technology, Faculty of Mechanical Engineering, TU Dortmund University, Leonhard-Euler-Straße 5, 44227 Dortmund, Germany
* Correspondence: ulrich.handge@tu-dortmund.de; Tel.: +49-231-755-8628

Supporting Information

A – Material Characterization

1. Effect of temperature on solubility

For water solubility tests on PVP K 30, cylindrical samples measuring 8 mm in diameter and 2.3 mm in thickness were prepared using the pressing device Vacuum MR Hei-End (MeltPrep GmbH). Samples, one at a time, were immersed in Millipore water measuring 60 mL in volume. Tests were carried out at temperatures 25 °C, 40 °C, 60 °C and 80 °C with constant stirring using a magnetic stirrer. Time taken for complete dissolution was measured.

The tests showed an increased rate of solubility with increase in temperature. The tests were performed in water, but it is assumed to have a similar effect on a dilute solution of NaOCl.

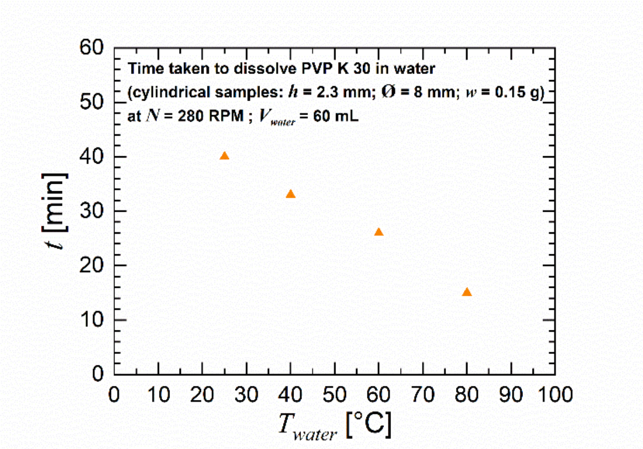

Figure S1. Time taken to dissolve PVP K 30 samples in water at various temperatures

2. Solubility tests in aqueous solution of NaOCl

Solubility tests were carried out for the blends H-8 and H-32 in aqueous solutions of NaOCl. Films of the respective blends of thickness 0.4 mm and width 21 mm were extruded using a twin screw extruder (Brabender, Duisburg, Germany) with a slit die.

Samples of length 25 mm were cut from the respective films. 0.1 wt% NaOCl solution with a pH value 11.5 was used because at this pH value, NaOCl has no interaction with PESU but dissolves PVP K 30 [35]. Two different temperatures, *i.e.* room temperature and 80 °C were chosen. Samples were inserted in the solution in a closed glass bottle at the given temperatures and were subjected to constant stirring using a magnetic stirrer for 48 hours. To wash out residual NaOCl, the samples were rinsed in decalcinated water at 35 °C for 10 minutes, Sodium disulfate ($Na_2S_2O_5$) at room temperature for 10 minutes and finally with decalcified water at 80 °C for 10 minutes. The effect of this treatment was observed using SEM.

Scanning electron micrographs showed no change on the surface of blend H-8 at both room temperatures and 80 °C. Blend H-32 did not undergo any visible change when treated with the NaOCl solution at room temperature. However, at 80 °C, uniform blind open pores appeared on the surface. These pores were of two sizes, smaller pores between 3 nm to 5 nm and larger pores between 20 nm to 50 nm. The depth of the larger pores was limited to approximately 50 nm. This confirms that although PVP is blended with PESU in single phase, it can be removed from the surface through post-treatment thus resulting in a porous structure. If the material is already porous on the inside, these surface pores could function as a selective layer thus implementable in ultrafiltration applications.

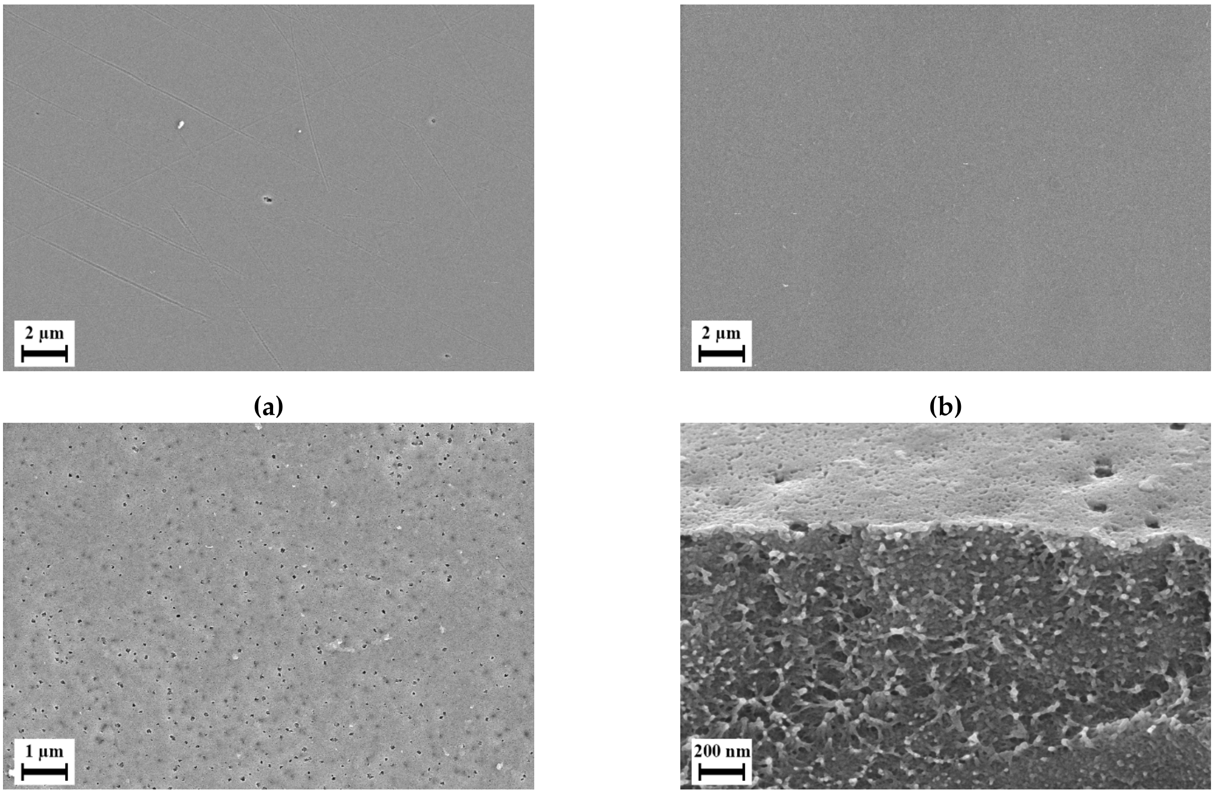

(c) (d)

Figure S2. Scanning electron micrographs of post-treated blends in NaOCl at various temperatures: **(a)** Surface of film of blend H-8 at 80 °C **(b)** Surface of film of blend H-32 at RT; **(c)** Surface of film of blend H-32 at 80 °C; **(d)** Cross-section of film of blend H-32 at 80 °C

3. Infrared spectrometry

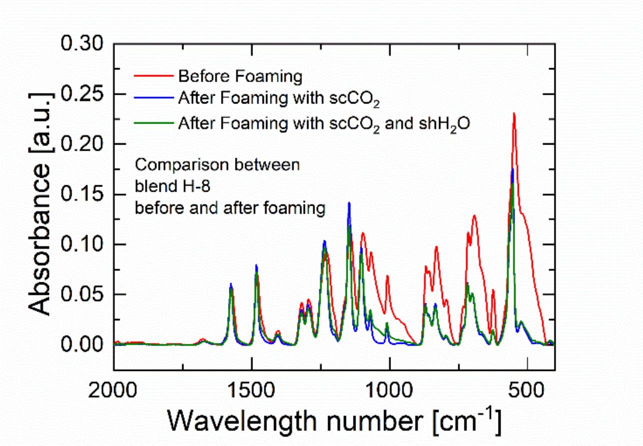

Figure S3. IR spectra of blend, non-foamed blend H-8 and after foaming with various foaming agents

Fourier transform infrared (FTIR) spectroscopy was carried out on some materials and their corresponding foams using a Bruker Alpha-P, platinum attenuated total reflection, equipped with diamond head (Bruker, Massachusetts, USA). Using FTIR spectroscopy, the chemical bonds in the material H-8, its foam using foaming agent CO_2 and its foam using foaming agent CO_2 and H_2O were analyzed. There is a significant difference between the material and the foams. The IR spectra of foams with both foaming agents show identical behavior. Due to foaming, a significant decrease in the sulfone (S=O) group, vinylene (C=C) group, and alkene (C=C) group can be seen. Exposure to loading temperature and sudden change in temperature after depressurization from loaded phase for foaming seem to cause the polymer system to undergo certain amount of degradation. Certain residual material was also observed on the inner lining of the reactor that could be wiped using organic solvents, indicating that they could be byproducts of these degradations.

4. DSC on unfoamed and foamed blend H-8

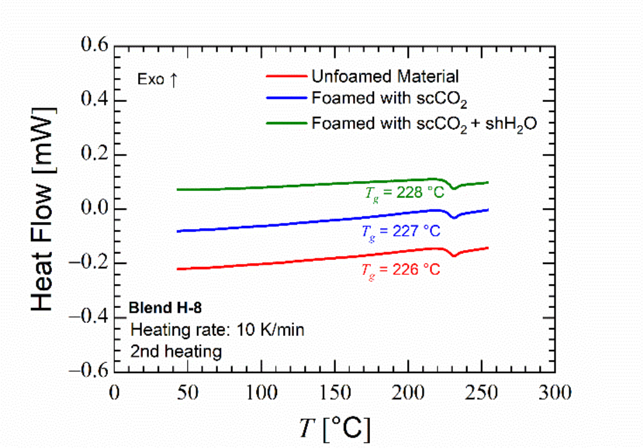

Figure S4. DSC 2nd heating curves of non-foamed blend H-8 and after foaming with various foaming agents

DSC measurements show minor and statistically insignificant differences between glass transition temperatures of material H-8 and its foams.

5. Rheological Analysis

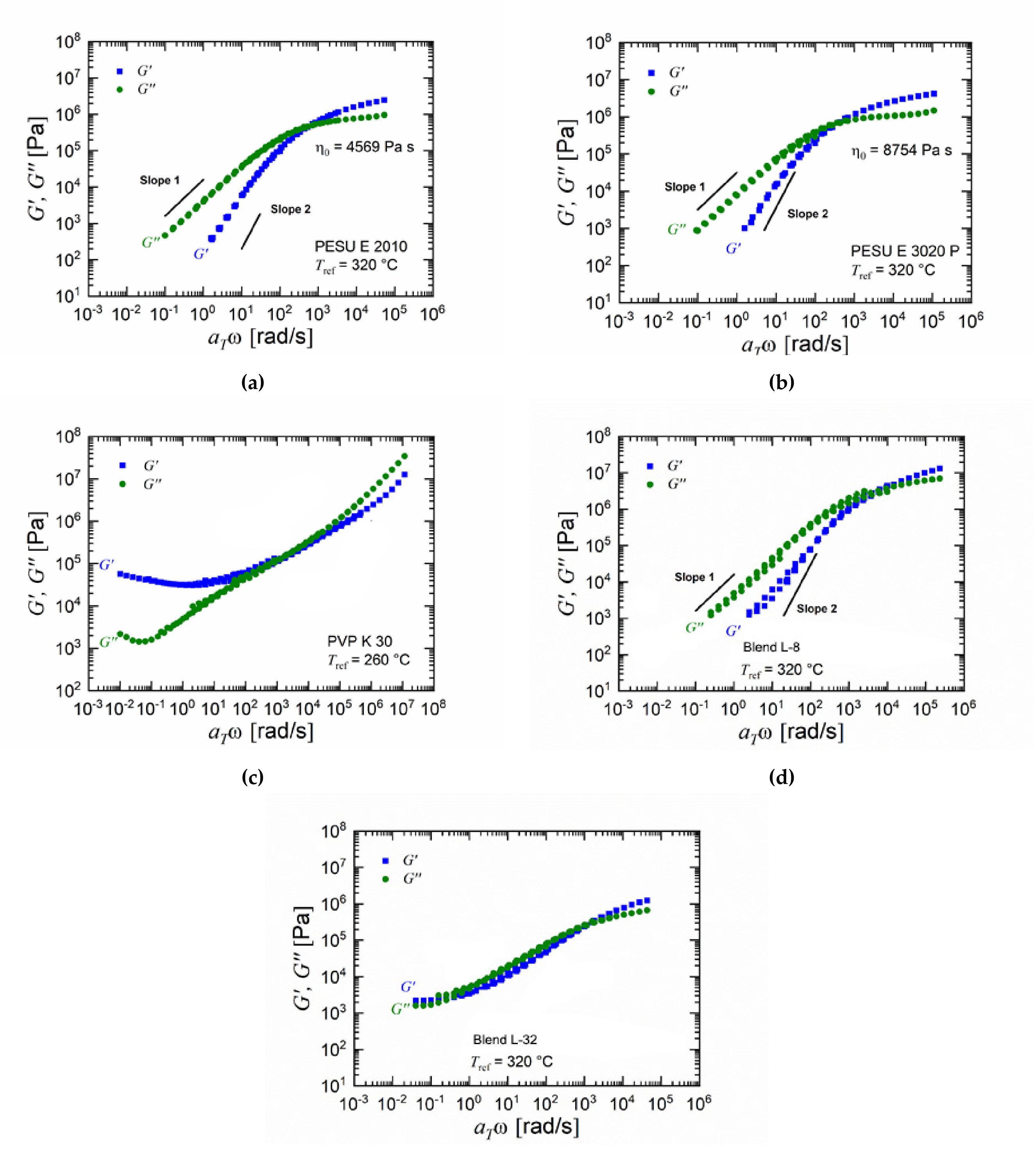

Figure S5. Master curves of selected materials from rheological analysis: **(a)** PESU E 2010; **(b)** PESU E 3020 P; **(c)** PVP K 30; **(d)** Blend L-8; **(e)** Blend L-32

6. Thermogravimetric analysis

Thermal gravimetric analysis (TGA) was carried out using a TGA 209 G1 220-11-0019-L Iris instrument (Netzsch, Selb, Germany). Measurements were carried out from 25 to 900 °C at a heating rate of 10 K min−1 in an argon atmosphere.

TGA provided an overview of their stability and decomposition temperature. In Figure 5(a), in argon environment, it can be observed that PESU E 3020 P starts degrading only at 475 °C, confirming a high thermal stability. PVP begins to degrade at temperatures which are higher than its glass transition temperature of 172 °C up to 410 °C and then rapidly decomposes up to 475 °C to a final relative mass of 4%. In the blends with 32% PVP K 30, this effect is seen slightly as the mass degrades up to 376 °C. In the blends with 8% PVP K 30, the polymer degrades up to 400 °C. Since blends with 32% PVP K 30 content show only 20% of mass reduction of pure PVP K 30, some sort of shielding effect caused by PESU can be assumed in these blends.

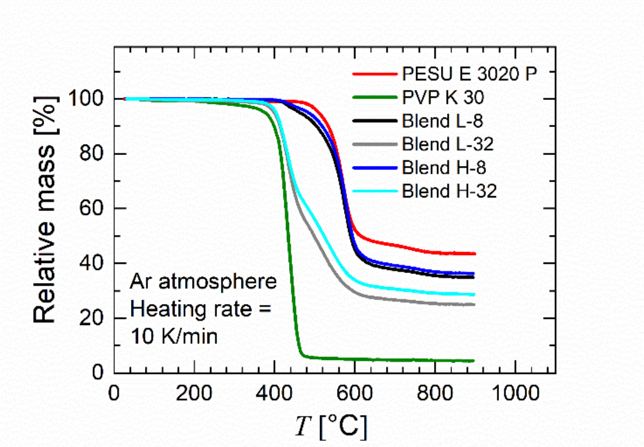

Figure S6. Results of thermogravimetric analysis for selected materials

B – Foams and membranes

1. Scanning electron micrographs of foams of blends foamed using CO_2 and H_2O versus foaming temperature

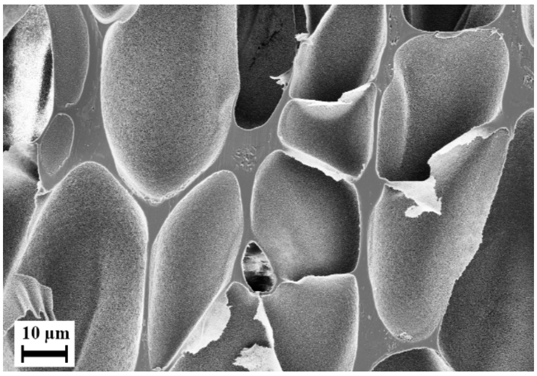

(a)

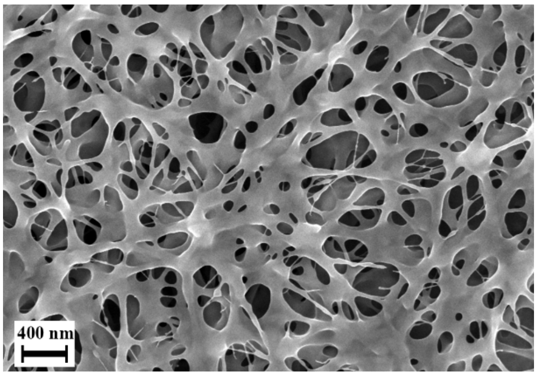

(b)

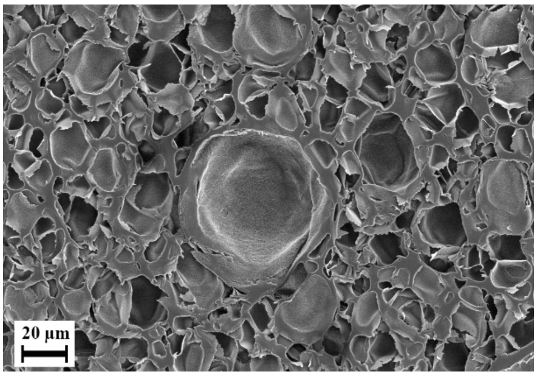

(c)

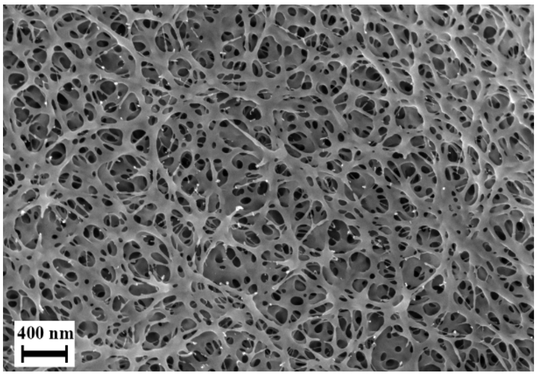

(d)

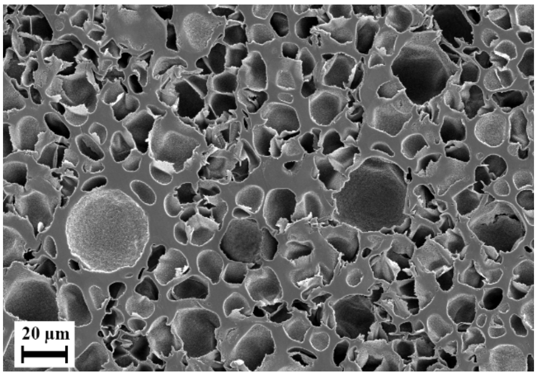

(e)

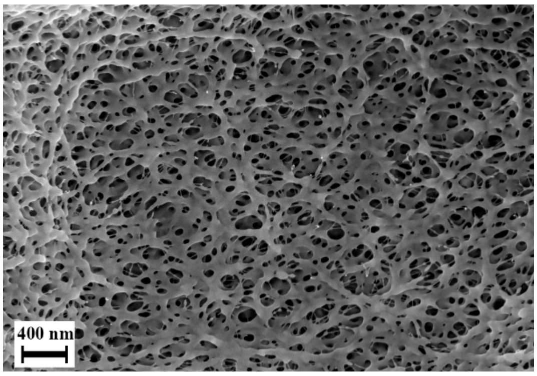

(f)

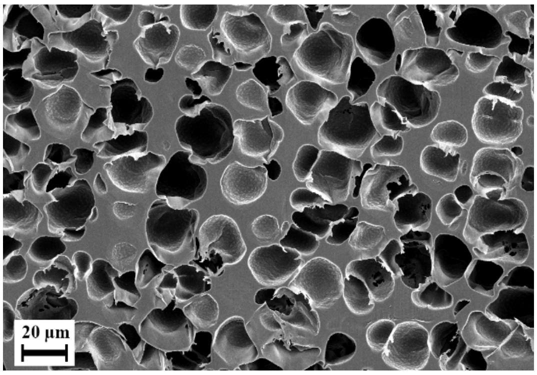

(g)

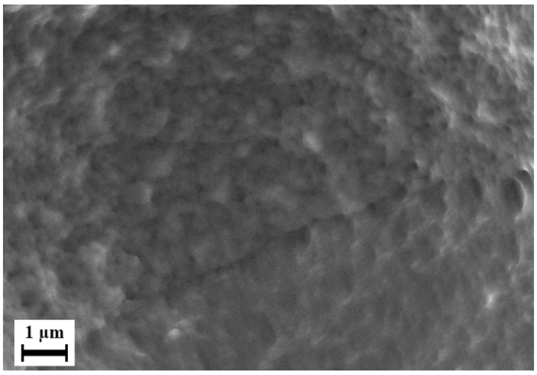

(h)

Figure S7. Scanning electron micrographs of blend L-8 foams manufactured using blowing agents CO_2 and H_2O at loading time 48 h, pressure 100 bar, loading temperature 150 °C, foaming time 100 s and various foaming temperatures: **(a), (b)** 210 °C; **(c), (d)** 230 °C; **(e), (f)** 250 °C; **(g), (h)** 270 °C

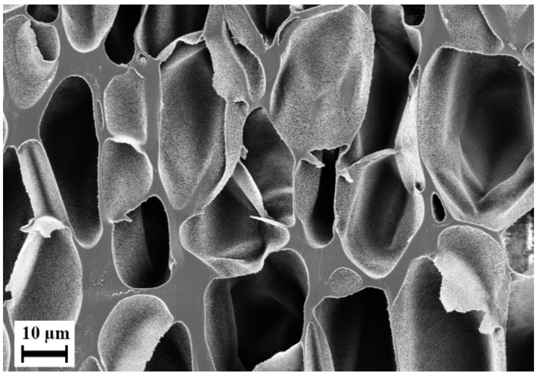

(a)

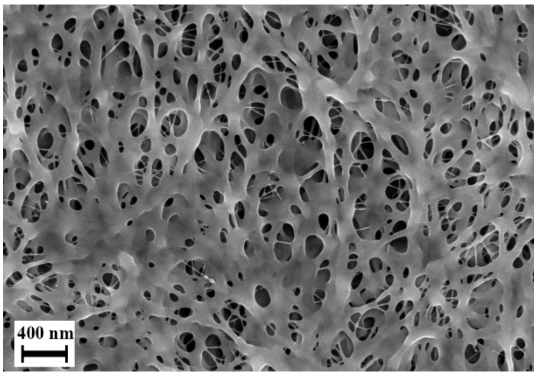

(b)

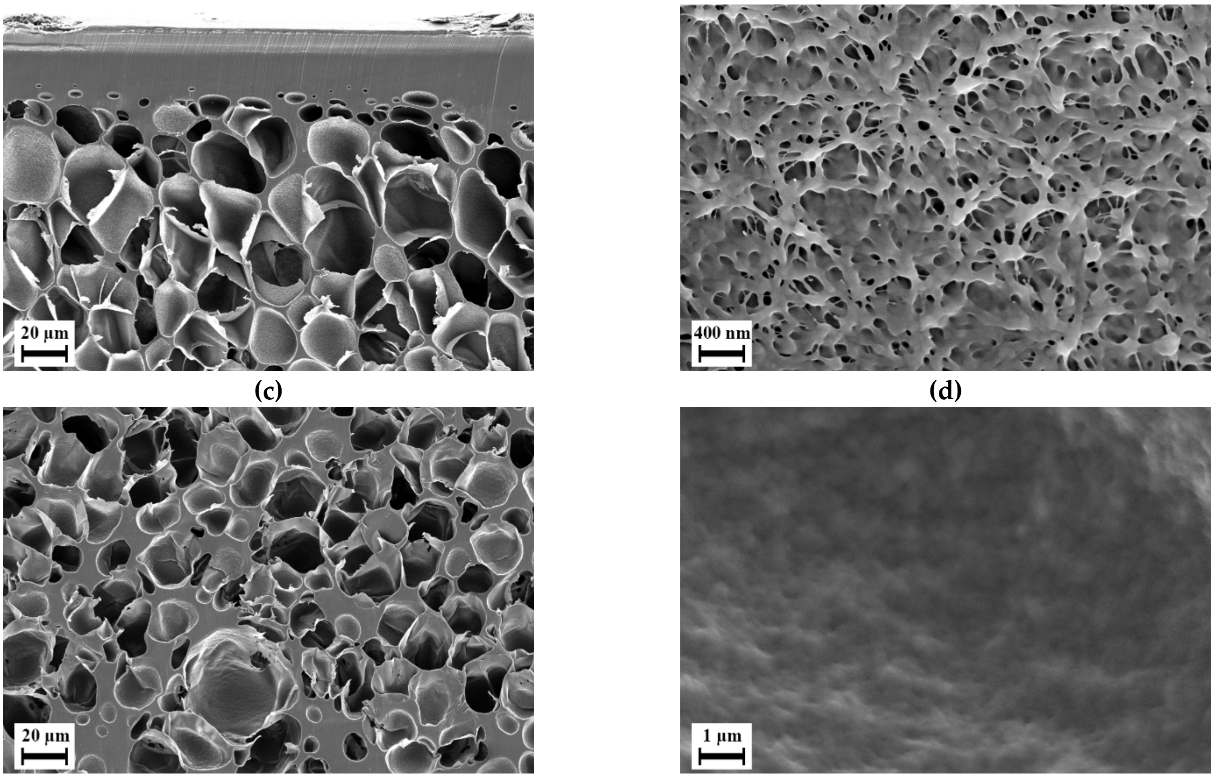

(c) (d)

(e) (f)

Figure S8. Scanning electron micrographs of blend H-8 foams manufactured using blowing agents CO_2 and H_2O at loading time 48 h, pressure 100 bar, loading temperature 150 °C, foaming time 100 s and various foaming temperatures: **(a), (b)** 210 °C; **(c), (d)** 250 °C; **(e), (f)** 270 °C

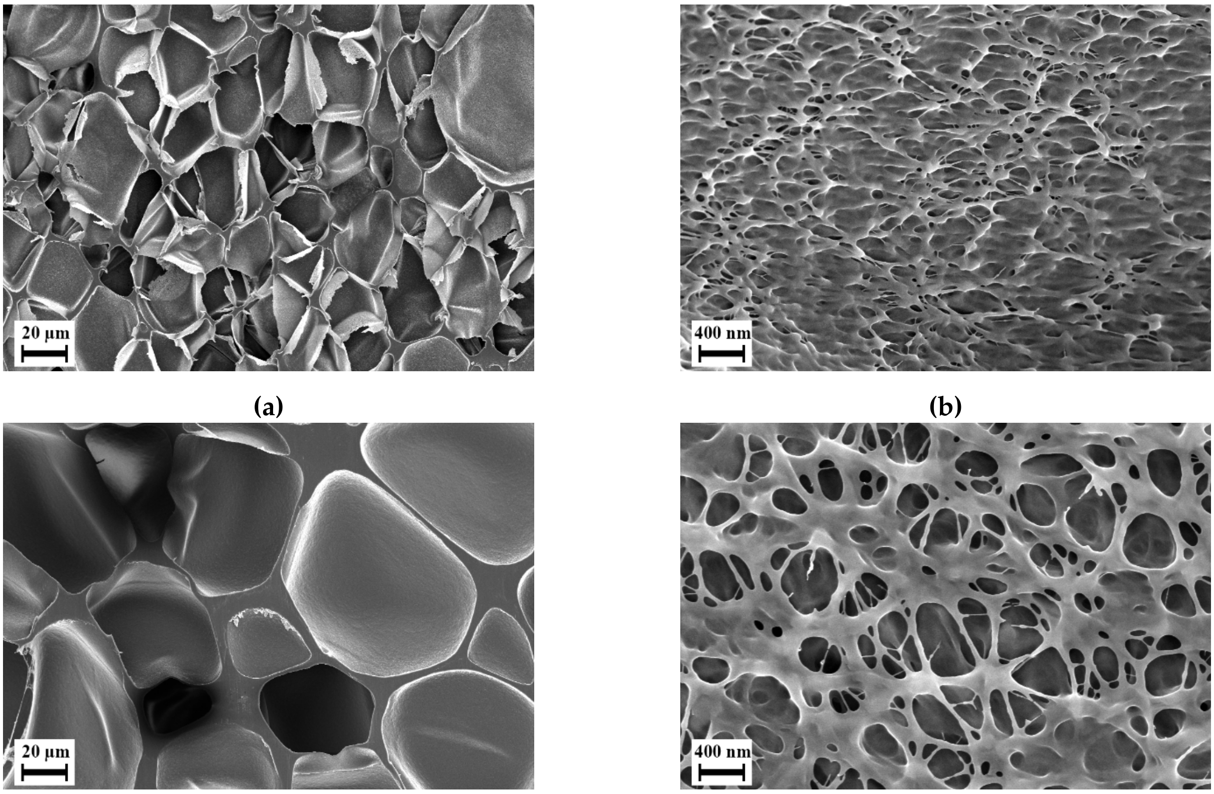

(a) (b) (c) (d)

Figure S9. Scanning electron micrographs of foams of blend H-8 at the loading time 48 h, pressure 100 bar, foaming temperature 230 °C, foaming time 100 s, the blowing agent CO_2 and H_2O and various loading temperatures: **(a)**, **(b)**: 125 °C; **(c)**, **(d)**: 175 °C.

2. Miscellaneous scanning electron micrographs

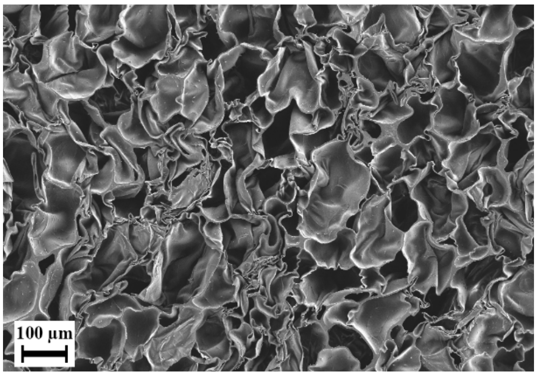

Figure S10. Scanning electron micrograph of collapsed PVP foam manufactured using blowing agent CO_2 at loading time 48 h, pressure 50 bar, loading temperature 150 °C, foaming time 100 s and foaming temperature 250 °C

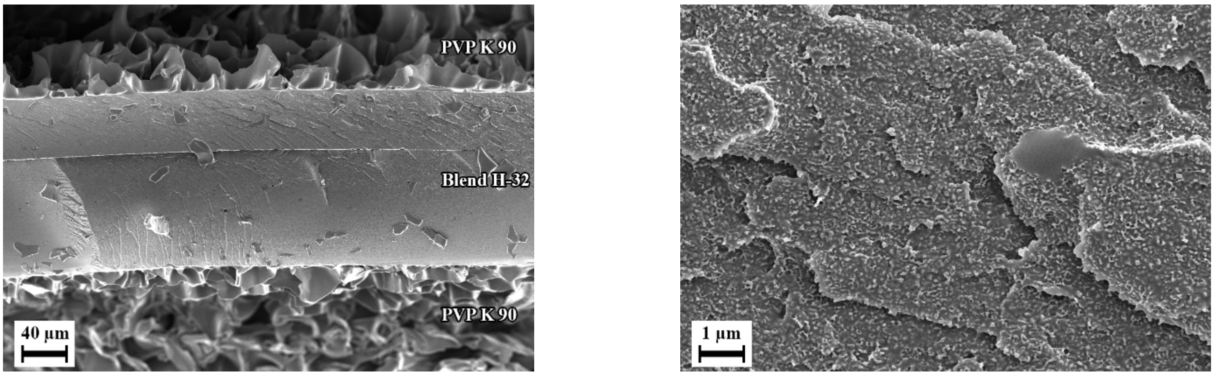

(a) (b)

Figure S11. Scanning electron micrographs of sandwich-type sample manufactured using method II with blend H-32: **(a)**, **(b)** after loading phase without subjecting to foaming temperatures (Similar settings and method used as Figure 12)

References

35. Dibrov, G.; Kagramanov, G.; Sudin, V.; Grushevenko, E.; Yushkin, A.; Volkov, A. Influence of sodium hypochlorite treatment on pore size distribution of polysulfone/polyvinylpyrrolidone membranes. Membranes 2020, *10*, 356. https://doi.org/10.3390/membranes10110356.
